# BMP-4 enhances epithelial mesenchymal transition and cancer stem cell properties of breast cancer cells via Notch signaling

**DOI:** 10.1038/s41598-019-48190-5

**Published:** 2019-08-13

**Authors:** Sanghyuk Choi, Jinyeong Yu, Aran Park, Maria Jose Dubon, Jungbeom Do, Youngjae Kim, Donghyun Nam, Jinok Noh, Ki-Sook Park

**Affiliations:** 1 Graduate School of Biotechnology, Kyung Hee University, Yongin, 17104 Korea; 2 Department of Biomedical Science and Technology, Graduate School, Kyung Hee University, Seoul, 02447 Korea; 3 East-West Medical Research Institute, Kyung Hee University, Seoul, 02447 Korea; 4 College of Medicine, Kyung Hee University, Seoul, 02447 Korea

**Keywords:** Cancer stem cells, Breast cancer

## Abstract

Bone morphogenetic protein (BMP) signaling and Notch signaling play important roles in tumorigenesis in various organs and tissues, including the breast. BMP-4 enhanced epithelial mesenchymal transition (EMT) and stem cell properties in both mammary epithelial cell line and breast carcinoma cell line. BMP-4 increased the expression of EMT biomarkers, such as fibronectin, laminin, N-cadherin, and Slug. BMP-4 also activated Notch signaling in these cells and increased the sphere forming efficiency of the non-transformed mammary epithelial cell line MCF-10A. In addition, BMP-4 upregulated the sphere forming efficiency, colony formation efficiency, and the expression of cancer stem cell markers, such as Nanog and CD44, in the breast carcinoma cell line MDA-MB-231. Inhibition of Notch signaling downregulated EMT and stem cell properties induced by BMP-4. Down-regulation of Smad4 using siRNA impaired the BMP-4-induced activation of Notch signaling, as well as the BMP-4-mediated EMT. These results suggest that EMT and stem cell properties are increased in mammary epithelial cells and breast cancer cells through the activation of Notch signaling in a Smad4-dependent manner in response to BMP-4.

## Introduction

Bone morphogenetic proteins (BMPs) are members of the transforming growth factor-β (TGF-β) superfamily. BMPs regulate various biological processes, such as proliferation, apoptosis, embryonic development, tumorigenesis, epithelial mesenchymal transition (EMT), and metastasis^1–5^. BMPs are expressed in breast cancer primary tumors, and their signaling pathways are activated in bone metastatic lesions found in breast cancer patients^2^. Although the roles of BMPs in breast cancer remain controversial^1^, the aberrant expression of BMPs in breast tumor is associated with both poor clinical outcomes and an increased frequency of disease recurrence^2,6^. One BMP family member, BMP-4, has been shown to be aberrantly expressed in breast cancer^2,6^. *In vitro*, BMP-4 upregulates EMT in mammary epithelial cells and breast tumor cells^7,8^. BMP-4 has also been shown to be necessary for the invasion of breast cancer cells, both *in vivo* and *in vitro*^9–11^.

BMP signal transduction occurs through heterodimers comprised of the BMP type 1 receptor and the type 2 receptor^4,12,13^. In response to binding BMPs, BMP type 1 receptors are phosphorylated by BMP type 2 receptors, and then become activated. The activated BMP type 1 receptors phosphorylate Smad1/5/9 proteins, which then form a complex with Smad4. This Smad complex enters the nucleus in order to regulate the expression of BMP downstream genes^12,13^. BMPs are able to transduce signals in either a Smad4 dependent (canonical signaling) or Smad4 independent (non-canonical signaling) manner. Following the activation of BMP receptors, various proteins such as JNK, Trb3, Src, and LIM kinase 1 mediate non-canonical signaling^14–16^.

Notch signaling is also critical for essential biological processes such as normal embryonic development, cell fate determination, and tumor formation^17–19^. Notch signaling is activated when transmembrane Notch ligands on one cell bind to transmembrane Notch receptors on another juxtaposed cell. In response to the binding, the transmembrane Notch receptors are cleaved by γ-secretase and the cleaved intracellular fragments derived from the Notch receptor are released intracellularly. These intracellular Notch fragments enter the nucleus where they act to regulate the expression of Notch downstream genes, such as Hey1 and Hes1^20,21^. In humans, there are four Notch receptors: Notch1, 2, 3, and 4 whose activities are controlled by Delta-like (Dll1, 3, and 4) and Jagged (Jagged-1 and -2) ligands^5^. The activation of Notch signaling plays important roles in the recurrence of dormant tumor cells, EMT, and cancer stem cell activity in breast cancer^22–24^. In human breast cancer, the expression of Notch1 and Jagged-1 is also significantly increased, and their enhanced expression is associated with poor survival, invasion, EMT, and bone metastasis^24–28^.

Along with BMPs, TGF-β1 is also a member of the transforming growth factor-β (TGF-β) superfamily. TGF-β1 induces EMT in epithelial cells in the mammary gland and promotes the progression of breast cancer^29–32^. Since it is known that EMT enhances the generation of cancer stem cells in human breast cancer^33^, the TGF-β1 dependent EMT enhances cancer stem cell activity and bone metastasis in breast cancer^34,35^. Moreover, it is also known that Notch signaling activated by TGF-β mediates EMT induced by TGF-β1^32^.

We have previously reported that BMP-4 induces EMT in the mammary epithelial cell line MCF-10A^8^. It is also well known that BMP-4 is required for EMT in breast cancer cells^7^. However, it is still not known whether EMT induced by BMP-4 is able to enhance stem cell properties both in mammary epithelial cells and breast cancer cells. It is also unknown as to whether Notch signaling mediates the BMP-4-induced EMT in the cells. Here, we examined the nature of BMP-4 signaling that mediates EMT in mammary epithelial cells and breast cancer cells.

## Results

### BMP-4 induces EMT and activates Notch signaling in the MCF-10A human mammary epithelial cell line

**Figure 1:**
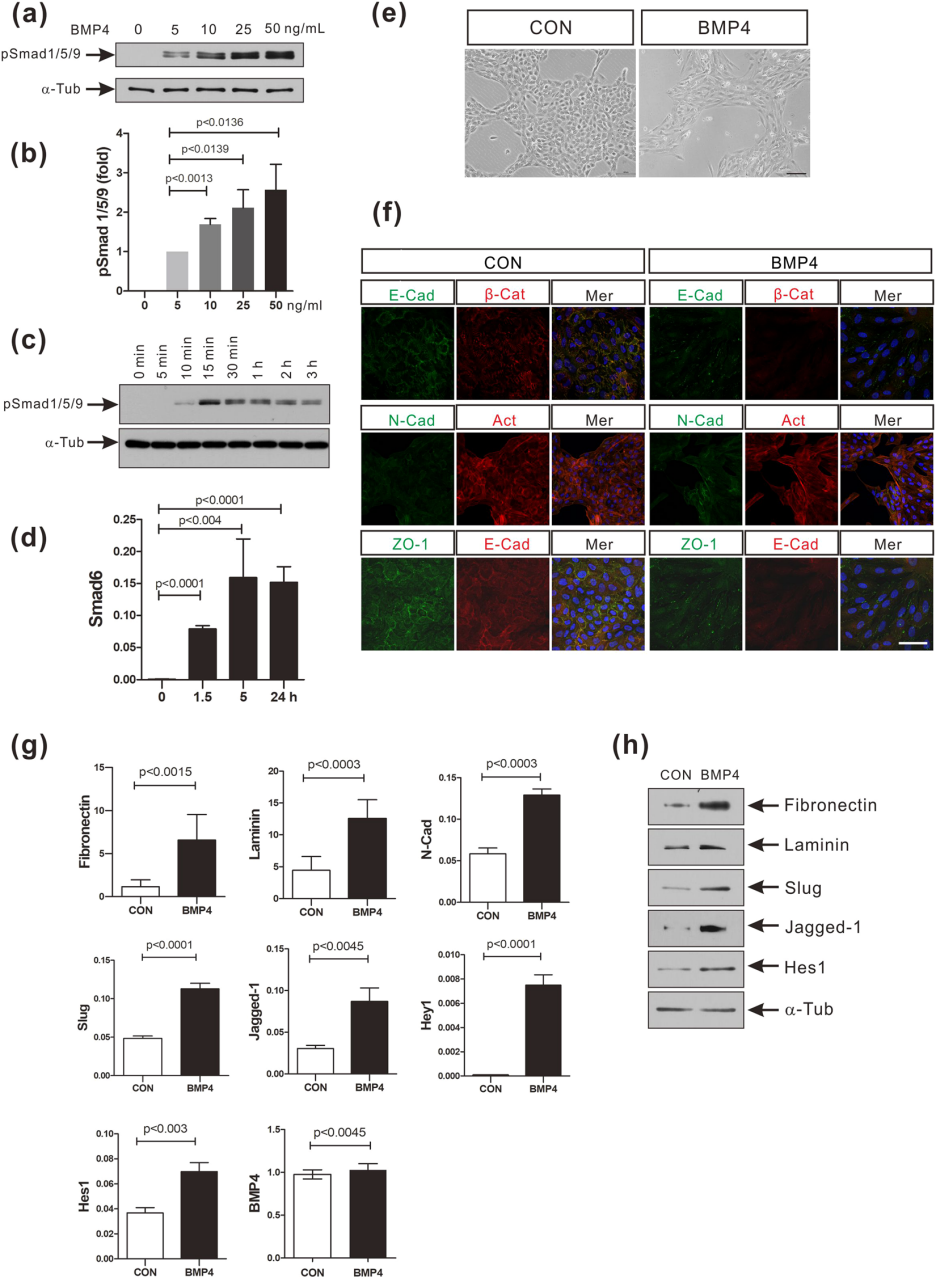
BMP-4 induces the EMT and the activation of Notch signaling in MCF-10A cells. (**a**) Western blot analysis of phosphorylated Smad1/5/9 proteins (pSmad1/5/9) in extracts of MCF-10A cells treated with BMP-4 (0, 5, 10, 25, 50 ng/ml). α-Tubulin was used as an internal control. The full-length blots/gels are presented in Supplementary Fig. 1a,b. (**b**) Densitometric analysis of western blot data from (**a**). Data are presented as the mean ± SD and *p*-values were calculated using Student’s *t*-test (3 independent experiments). (**c**) Western blot analysis of pSmad1/5/9 in extracts of MCF-10A cells treated with BMP-4 (50 ng/ml) for the indicated times. α-Tubulin was used as an internal control. The full-length blots are presented in Supplementary Fig. 1c,d. (**d**) Real-time PCR analysis of Smad6 expression in MCF-10A cells treated with BMP-4 (50 ng/ml) for the indicated times. Human ribosomal protein S9 (RPS9) was used as an internal control. Data are presented as the mean ± SD and *p*-values were calculated using a Student’s *t*-test (3 independent experiments). (**e**) Representative images of MCF-10A cells treated with BMP-4 (50 ng/ml) or vehicle (CON) for 24 hours. Scale bar: 100 µm. (**f**) Immunocytochemistry of MCF-10A cells treated with BMP-4 (50 ng/ml) or vehicle (CON) for 24 hours. E-cadherin (E-cad) was stained either green or red. ZO-1 or N-cadherin (N-cad) were stained green. β-Catenin (β-Cat) was stained red. Actin (Act) was stained red with phalloidin and nuclei were stained blue with DAPI. Mer, merged images. Scale bar: 50 µm. (**g**) Real-time PCR analysis of EMT-associated genes and Notch target genes in MCF-10A cells treated with BMP-4 (50 ng/ml) or vehicle (CON) for 24 hours. Endogenous BMP-4 expression was analyzed at 5 hours post treatment of BMP-4. Human ribosomal protein S9 (RPS9) was used as an internal control. Data are presented as the mean ± SD and *p*-values were calculated using a Student’s *t*-test (3 independent experiments). (**h**) Western blot analysis of EMT-associated proteins and Notch target proteins in the MCF-10A cells treated with BMP-4 (50 ng/ml) or vehicle (CON) for 24 hours. The full-length blots are presented in Supplementary Fig. 4.

**Figure 2:**
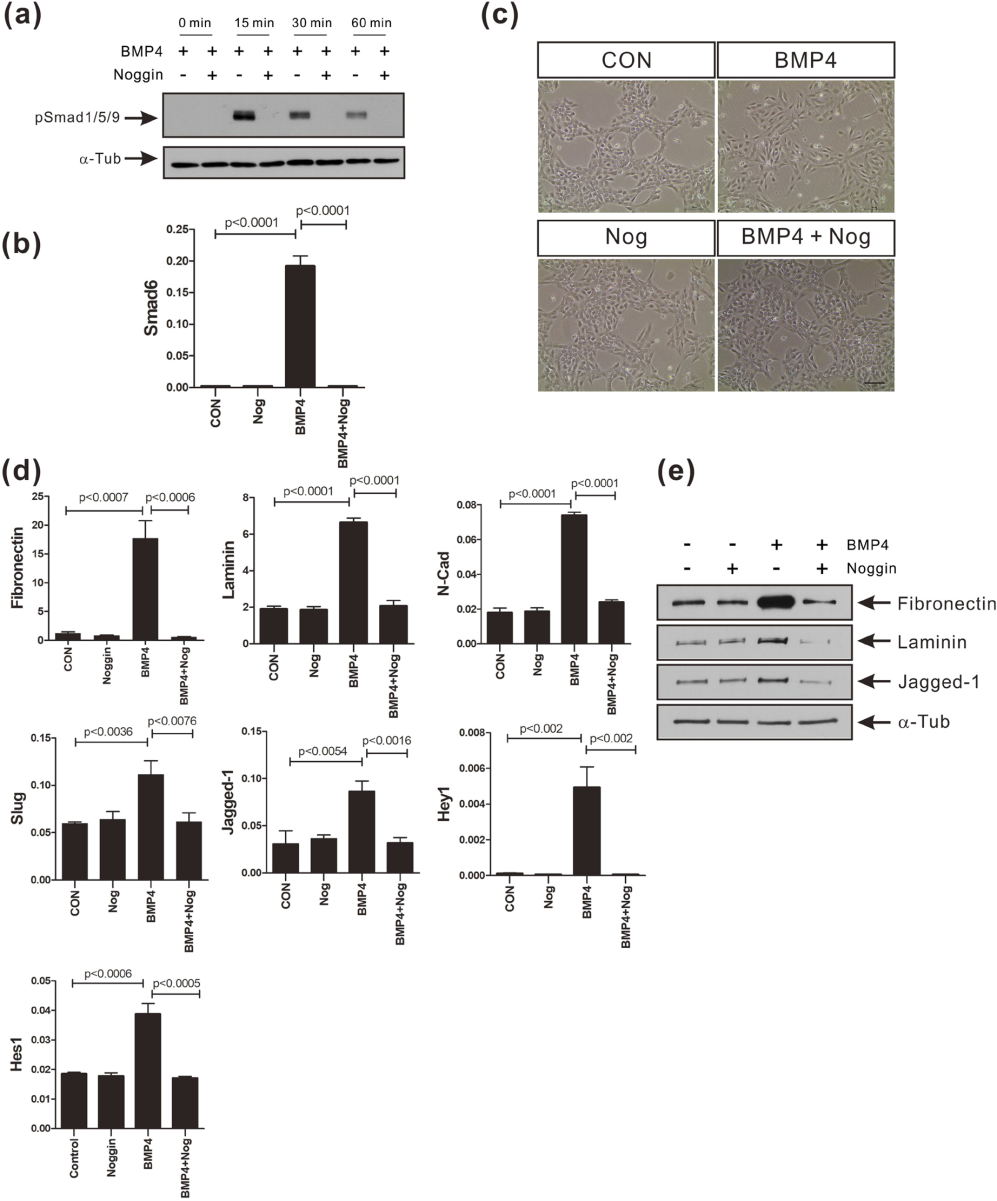
Noggin inhibits the BMP-4-induced EMT and increases the expression of Notch target genes in MCF-10A cells. (**a**) Western blot analysis of phosphorylated Smad1/5/9 proteins (pSmad1/5/9) in extracts of MCF-10A cells treated with 200 ng/ml Noggin for 30 minutes prior to BMP-4 (50 ng/ml) treatment for the indicated times. α-Tubulin was used as an internal control. The full-length blots are presented in Supplementary Fig. 5. (**b**) Real-time PCR analysis of Smad6 expression in MCF-10A cells treated with 200 ng/ml Noggin for 30 minutes prior to BMP-4 (50 ng/ml) treatment for 24 hours. Human ribosomal protein S9 (RPS9) was used as an internal control. (**c**) Representative images of MCF-10A cells treated with 200 ng/ml Noggin for 30 minutes prior to BMP-4 (50 ng/ml) treatment for 24 hours. Scale bar: 100 µm. (**d**) Real-time PCR analysis of EMT-associated genes and Notch target genes in MCF-10A cells treated with 200 ng/ml Noggin for 30 minutes prior to BMP-4 (50 ng/ml) treatment for 24 hours. Human ribosomal protein S9 (RPS9) was used as an internal control. Data are presented as the mean ± SD and *p*-values were calculated using the Student’s *t*-test (3 independent experiments). (**e**) Western blot analysis of fibronectin, laminin, and Jagged-1 in MCF-10A cells treated with 200 ng/ml Noggin for 30 minutes prior to BMP-4 (50 ng/ml) treatment for 24 hours. α-Tubulin was used as an internal control. The full-length blots are presented in Supplementary Fig. 6.

As we have reported previously, BMP-4 activated the canonical BMP signaling pathway and induced the epithelial mesenchymal transition (EMT) in the human mammary epithelial cell line MCF-10A (Fig. 1). BMP-4 induced phosphorylation of Smad1/5/9 in a dose-dependent manner, with maximal phosphorylation of Smad1/5/9 being observed at 50 ng/ml BMP-4 (Fig. 1a,b). BMP-4 (50 ng/ml) transiently induced the phosphorylation of Smad1/5/9, with the phosphorylation of Smad1/5/9 gradually decreasing at longer time points (Fig. 1c). Smad6 is an inhibitory Smad protein that terminates BMP signaling by blocking either the interaction between Smad1 and Smad4 or the phosphorylation of Smad1^36,37^. Smad6 is induced as part of a negative feedback mechanism in response to BMP treatment, and this process is dependent on the BMP activation of Smad1/5 and Smad4^38^. The level of Smad6 mRNA was significantly increased 1.5 hours after the MCF-10A cells were treated with 50 ng/ml of BMP-4 (Fig. 1d) and consequently, the expression of Smad6 protein also increased (Supplementary Fig. 2). MCF-10A cells also underwent EMT in response to BMP-4 (Fig. 1e–h). At 24 hours after BMP-4 treatment, the cells had adopted a mesenchymal morphology (Fig. 1e). BMP-4-treated MCF-10A cells also expressed less E-cadherin, and more N-cadherin than that by control cells (Fig. 1f). The expression of ZO-1, a tight junction-related protein was also downregulated in MCF-10A cells after BMP-4 treatment (Fig. 1f). The mRNA levels of various EMT biomarkers, particularly fibronectin and laminin, were also found to be increased in BMP-4-treated MCF-10A cells (Fig. 1g). The mRNA levels of N-cadherin and Slug were also upregulated following the BMP-4 treatment, compared to the control cells (Fig. 1g). BMP-4 also activated the Notch signaling pathway which is critical for TGF-β-mediated EMT in breast cancer cells and mouse mammary gland epithelial cells^24,32^. Compared to the control cells, BMP-4 induced a 2.8-fold increase in the expression of Jagged-1 and also significantly increased the expression of the Notch downstream genes, Hey1 and Hes1 (Fig. 1g). The increase in mRNA levels of EMT markers, Jagged-1, Hey1, and Hes1 was confirmed with experiments using two different internal control genes (ribosomal protein S9 gene and glyceraldehyde 3-phosphate dehydrogenase gene) (Supplementary Fig. 3). However, the endogenous BMP-4 expression was not altered in response to BMP-4 treatment (Fig. 1g). Additionally, we observed BMP-4-mediated increase in the expression of fibronectin, laminin, Slug, Jagged-1, and Hes1 proteins (Fig. 1h). Noggin binds to and inactivates BMP-4^39,40^. Noggin not only inhibited the phosphorylation of Smad1/5/9 and the induction of the expression of Smad6 (Fig. 2a,b), but also blocked the induction of EMT in response to BMP-4 (Fig. 2c–e). More importantly, Noggin blocked the activation of Notch signaling mediated by BMP-4 (Fig. 2d,e). Taken together, these data support the notion that BMP-4 induces EMT and the activation of Notch signaling in the human mammary epithelial cell line MCF-10A.

### Notch signaling mediated EMT is induced by BMP-4

**Figure 3:**
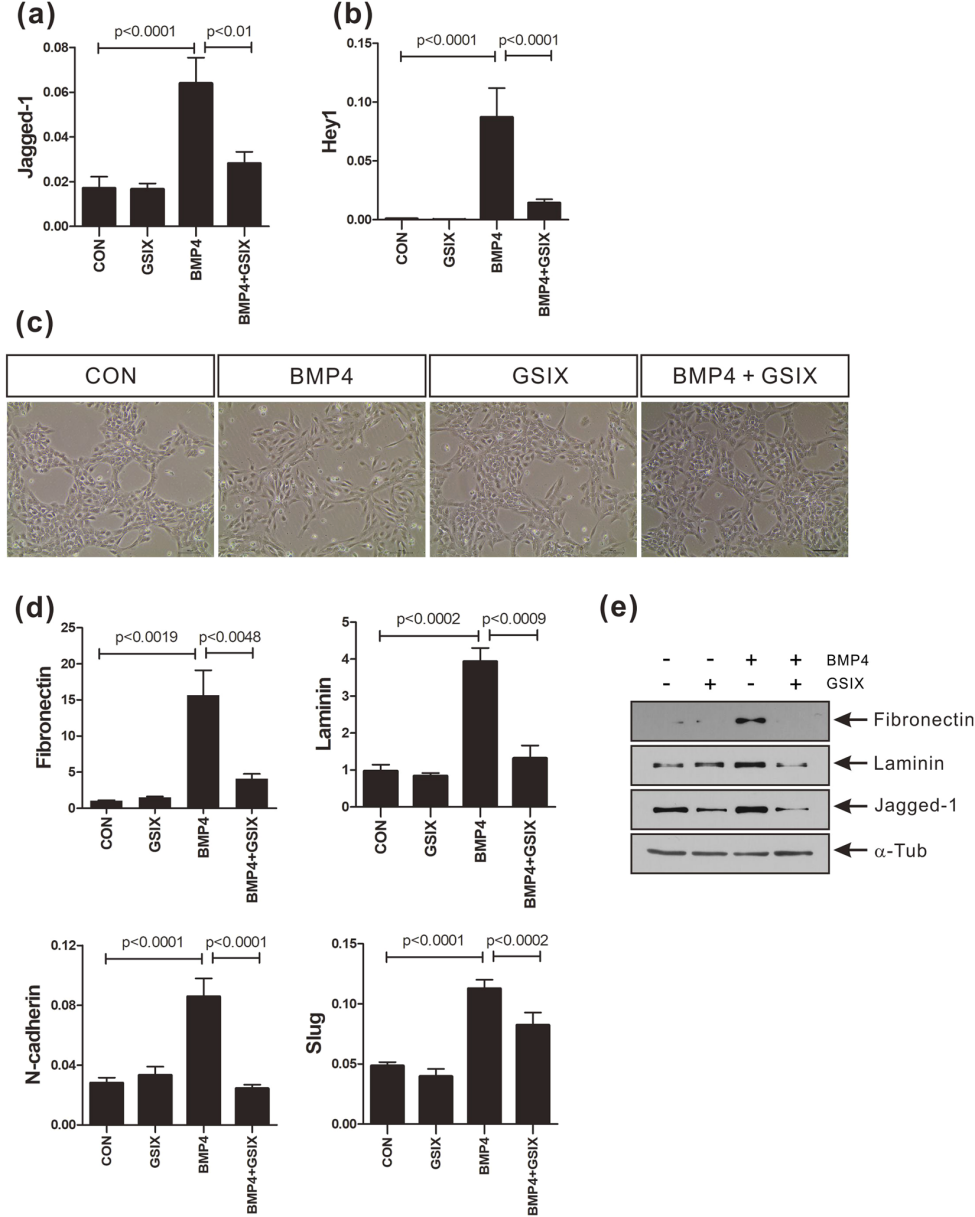
γ-Secretase inhibitor prevents the BMP-4-induced EMT and the increase in expression of Notch target genes in MCF-10A cells. (**a**,**b**) Real-time PCR analysis of Jagged-1 or Hey1 in MCF-10A cells treated with γ-secretase inhibitor X (GSIX; 5 μM) for 30 minutes prior to BMP-4 (50 ng/ml) treatment for 24 hours. Human ribosomal protein S9 (RPS9) was used as an internal control. (**c**) Representative images of MCF-10A cells treated with GSIX (5 μM) for 30 minutes prior to BMP-4 (50 ng/ml) treatment for 24 hours. Scale bar: 100 µm. (**d**) Real-time PCR analysis of fibronectin, laminin, N-cadherin, and Slug genes in MCF-10A cells treated with GSIX (5 μM) for 30 minutes prior to BMP-4 (50 ng/ml) treatment for 24 hours. Human ribosomal protein S9 (RPS9) was used as an internal control. Data are presented as the mean ± SD and *p*-values were calculated using a Student’s *t*-test (3 independent experiments). (**e**) Western blot analysis of fibronectin, laminin, and Jagged-1 in MCF-10A cells treated with GSIX (5 μM) for 30 minutes prior to BMP-4 (50 ng/ml) treatment for 24 hours. α-Tubulin was used as an internal control. The full-length blots are presented in Supplementary Fig. 7.

Next, we examined whether the Notch signaling activated by BMP-4 is necessary for the induction of EMT in MCF-10A cells. The cleavage of Notch receptors by γ-secretase is required for the activation of Notch signaling^21^. Pretreatment with a γ-secretase inhibitor X (GSIX, 5 μM) impaired the BMP-4 mediated activation of Notch signaling in MCF-10A cells. The BMP-4-mediated increase in the expression of Jagged-1 and Hey1 was blocked by pretreatment with GSIX prior to BMP-4 treatment (Fig. 3a,b). GSIX also inhibited EMT in response to BMP-4, and reversed the mesenchymal-like cell morphology induced by BMP-4 treatment (Fig. 3c). In addition, the BMP-4-mediated increase in the expression of fibronectin, laminin, N-cadherin, and Slug was inhibited by pretreatment with GSIX (Fig. 3d,e). These results suggest that EMT induced by BMP-4 is mediated through the activation of Notch signaling by BMP-4.

### BMP-4 induces the EMT and the activation of Notch signaling in a Smad4 dependent manner

**Figure 4:**
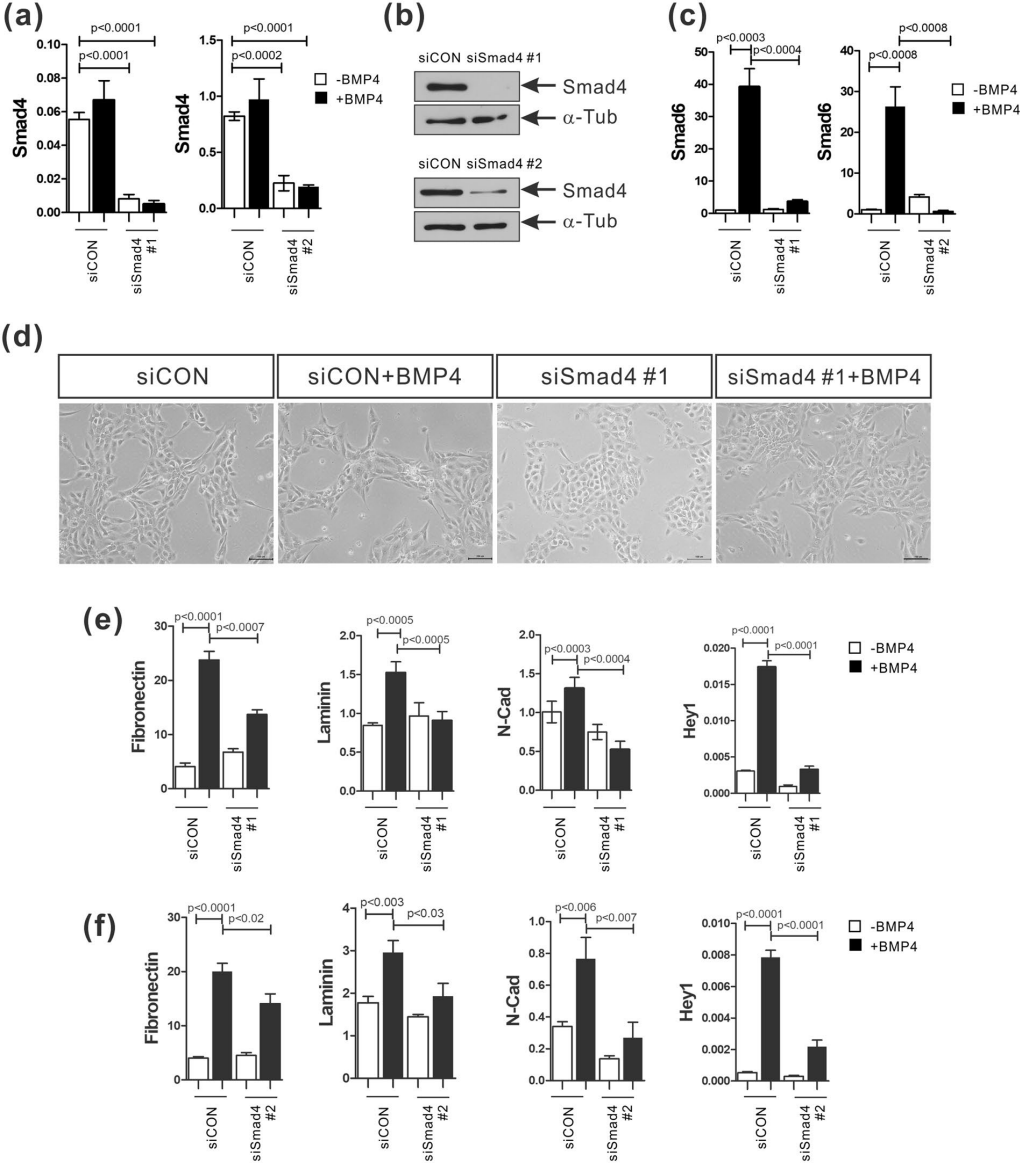
Smad4 knockdown prevents the BMP-4-induced EMT and the increase in expression of Notch target genes in MCF-10A cells. (**a,b**) mRNA and protein levels of Smad4 in MCF-10A cells transfected with Smad4 siRNAs (siSmad4 #1 or siSmad4 #2) or a control siRNA (siCON). Human ribosomal protein S9 (RPS9) was used as an internal control for real-time PCR and α-tubulin was used as an internal control for Western blot analysis. The full-length blots are presented in Supplementary Fig. 8. (**c**) Real-time PCR to analyze Smad6 mRNA expression in MCF-10A cells transfected with Smad4 siRNAs (siSmad4 #1 or siSmad4 #2) or a control siRNA (siCON). (**d**) Representative images of MCF-10A cells transfected with a Smad4 siRNA (siSmad4 #1) or a control siRNA (siCON) prior to BMP-4 (50 ng/ml) treatment for 24 hours. Scale bar: 100 µm. (**e,f**) Real-time PCR analysis of fibronectin, laminin, N-cadherin, and Hey1 mRNA expression in MCF-10A cells transfected with Smad4 siRNAs (siSmad4#1 in **e** or siSmad4#2 in **f**) or a control siRNA (siCON) prior to BMP-4 (50 ng/ml) treatment for 24 hours. Human ribosomal protein S9 (RPS9) was used as an internal control. Data are presented as the mean ± SD and *p*-values were calculated using a Student’s *t*-test (3 independent experiments).

To investigate whether BMP-4 canonically (Smad4 dependent) or non-canonically (Smad4 independent) induces EMT and Notch signaling, we created a knockdown of Smad4 by transfecting with two sets of Smad4-targeted siRNA. Compared to the cells transfected with control siRNA, the cells transfected with Smad4-targeted siRNA #1 or siRNA #2 had decreased Smad4 mRNA and protein levels (Fig. 4a,b). Smad4 knockdown also abolished the increase in Smad6 mRNA levels achieved in response to BMP-4, suggesting activation of the BMP-4 canonical signaling pathway (Fig. 4c). In accordance with these data, Smad4 knockdown also reversed the acquisition of the mesenchymal-like cell morphology induced by BMP-4 (Fig. 4d). More importantly, Smad4 knockdown inhibited the induction of Hey1 as well as fibronectin, laminin, and N-cadherin in response to BMP-4 (Fig. 4e,f). Thus, these results suggest that BMP-4 canonically activates the Notch signaling pathway that mediates the EMT in MCF-10A cells.

### BMP-4 promotes stem cell activity in MCF-10A cells via Notch signaling

**Figure 5:**
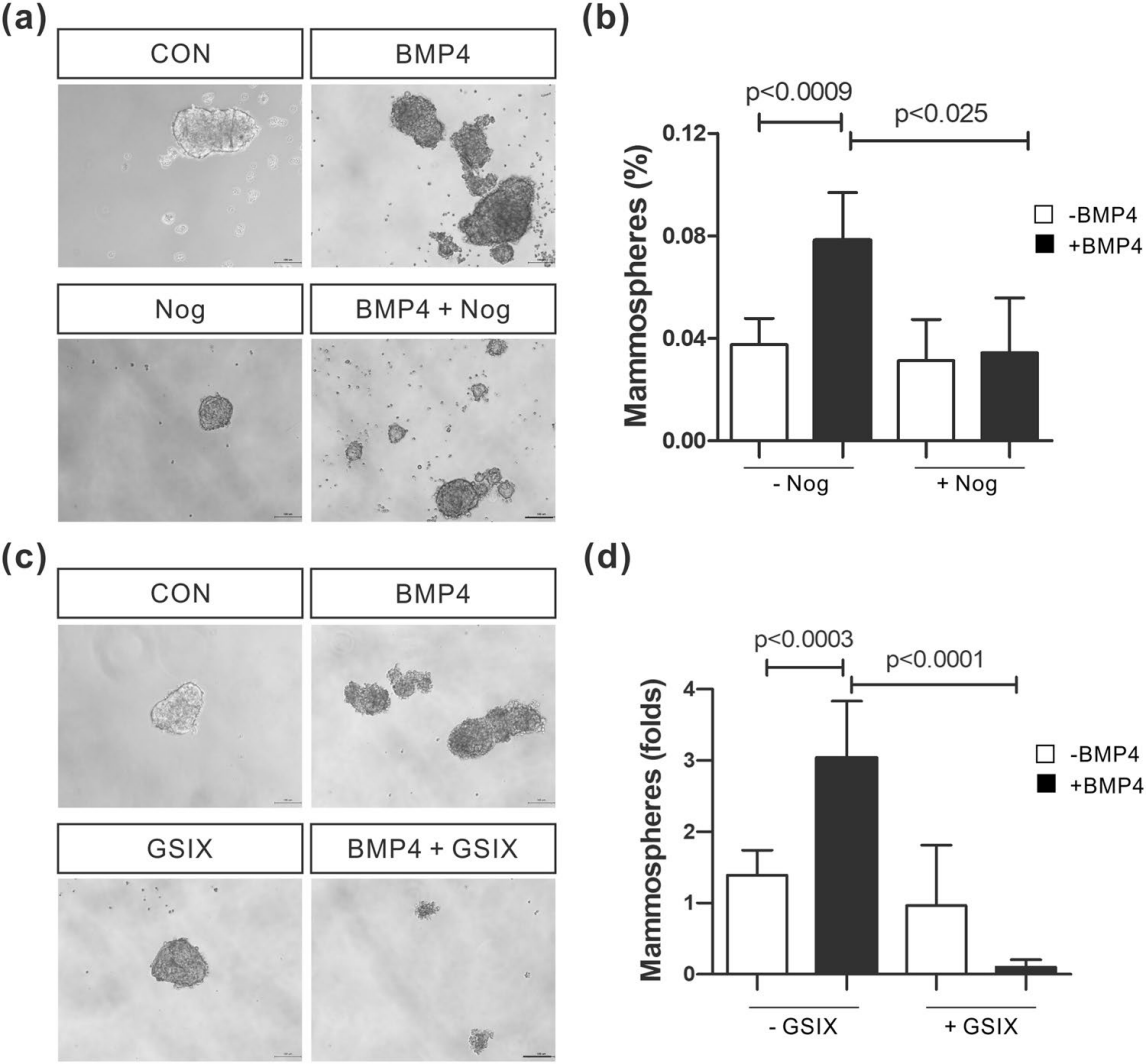
BMP-4 enhances the mammosphere formation ability of MCF-10A cells. (**a**) Mammospheres formed by MCF-10A cells treated with BMP-4 (50 ng/ml) and/or Noggin (200 ng/ml) for 9 days. Scale bar: 100 µm. (**b**) Quantitative data showing the fold difference in the mammosphere forming ability of MCF-10A cells treated with BMP-4 and/or Noggin. (**c**) Mammospheres formed by MCF-10A cells treated with BMP-4 (50 ng/ml) and/or γ-secretase inhibitor X (GSIX; 5 μM) for 9 days. Scale bar: 100 µm. (**d**) Quantitative data showing the fold difference in the mammosphere forming ability of MCF-10A cells treated with BMP-4 and/or GSIX. Data are presented as the mean ± SD and *p*-values were calculated using a Student’s *t*-test (3 independent experiments).

Stem cells in mouse mammary epithelial cells and cancer stem cells in human breast cancer express EMT-associated biomarkers, such as N-cadherin, Slug, and Twist^33^. Moreover, EMT enhances the generation of cancer stem cells in human breast cancer^33^. The capacity for mammosphere formation is increased in stem cells isolated from mammary epithelia and breast cancer, compared to non-stem cells^41,42^. Thus, a mammosphere formation assay was performed to investigate whether the Smad4-dependent, Notch signaling-mediated, EMT affects stem cell activity in these MCF-10A human mammary epithelial cells. A 2-fold increase in mammosphere formation ability was observed in BMP-4-treated MCF-10A cells, compared to control cells (Fig. 5a,b; 0.038 ± 0.0005 [n = 4 wells] vs. 0.078 ± 0.0009 [n = 4 wells], respectively; p < 0.0009). Pretreatment with Noggin inhibited the BMP-4-mediated increase in mammosphere forming ability (Fig. 5a,b). Inactivation of Notch signaling using pretreatment with a γ-secretase inhibitor (GSIX) also downregulated the capacity of cells to form mammospheres (Fig. 5c,d). Therefore, Notch signaling activated through BMP-4 is required to enhance the stem cell capacity of mammary epithelial cells, as well as for the induction of EMT.

### BMP-4 promotes the EMT, Notch signaling, and the cancer stem cell capacity of breast cancer cell

**Figure 6:**
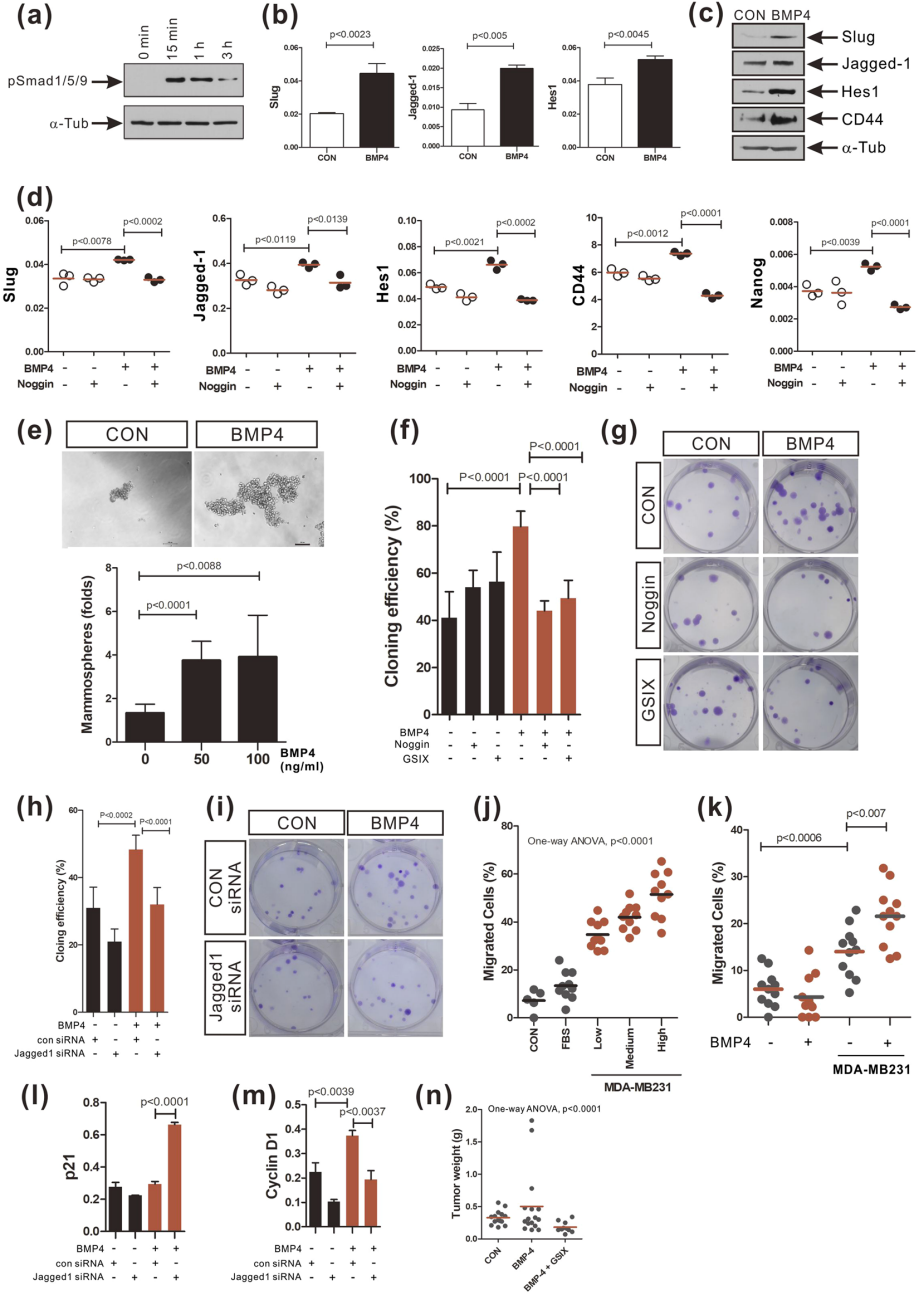
BMP-4 enhances EMT, Notch signaling, cancer stem cell capacity, and tumorigenesis in MDA-MB-231 cells. (**a**) Phosphorylated Smad1/5/9 proteins (pSmad1/5/9) detected in MDA-MB-231 cells treated BMP-4 (50 ng/ml) for the indicated time durations. The full-length blots have been presented in Supplementary Fig. 9. (**b,c**) Real-time PCR analysis (**b**) and Western blot analysis (**c**) of the expression of Slug, Jagged-1, Hes1, and CD44 in MDA-MB-231 cells treated with BMP-4 (50 ng/ml) for 72 hours. The full-length blots have been presented in Supplementary Fig. 10. (**d**) Real-time PCR analysis of the expression of Slug, Jagged-1, Hes1, CD44, and Nanog in MDA-MB-231 cells treated with Noggin for 30 minutes prior to BMP-4 treatment for 72 hours. The red lines indicate the mean values. (**e**) Mammospheres formed by MDA-MB-231 cells treated with BMP-4 (0, 50 or 100 ng/ml) for 10 days (Scale bar: 100 µm, n = 6 wells, 2 independent experiments). (**f,g**) Colony formation efficiency of MDA-MB-231 cells treated with Noggin or GSIX for 30 minutes prior to BMP-4 treatment for 72 hours. (n = 6 wells, 3 independent experiments). (**h,i**) Colony formation efficiency of MDA-MB-231 cells transfected with a Jagged-1 siRNA or a control siRNA prior to BMP-4 treatment for 72 hours (n = 6 wells, 3 independent experiments). (**j**) Migration of bone marrow-derived mesenchymal stem cells toward indirectly co-cultured MDA-MB-231 cells. For the co-culture, MDA-MB-231 cells were cultured at 3 different densities (CON; no cells, Low; 1 × 10^4^ cells/cm^2^, Medium; 2 × 10^4^ cells/cm^2^, High; 3 × 10^4^ cells/cm^2^). MDA-MB-231 complete culture media including 10% FBS was used as a positive control (2 independent experiments). (**k**) Migration of bone marrow-derived mesenchymal stem cells toward MDA-MB-231 cells pretreated or not with BMP-4 for 72 hours (2 independent experiments). (**l,m**) The expression of p21 (**l**) and cyclin D1 (**m**) in MDA-MB-231 cells transfected with a Jagged-1 siRNA or a control siRNA prior to BMP-4 treatment for 72 hours. (**n**) Growth of the MDA-MB-231 xenografts in nude mice (2 independent experiments, 10~16 mice/group; p < 0.0001, ANOVA).

Next, following the increase in EMT, we examined whether BMP-4 is able to enhance the stem cell properties of the human breast cancer cell line MDA-MB-231, as well as it did in MCF-10A cells. BMP-4 (50 ng/ml) transiently increased the phosphorylation of Smad1/5/9 (Fig. 6a). Slug expression was upregulated in MDA-MB-231 cells in response to 50 ng/ml BMP-4 (Fig. 6b–d). The expression levels of breast cancer stem cell markers, such as CD44 and Nanog^33,43,44^, as well as the expression levels of Jagged-1 and Hes1, were increased in BMP-4-treated MDA-MB-231 cells, compared to control cells (Fig. 6b–d). Noggin pretreatment also impaired the BMP-4-induced increases in the expression levels of Slug, Jagged-1, Hes1, CD44, and Nanog (Fig. 6d). Since BMP-4 increased the expression of cancer stem cell markers in MDA-MB-231 cells, we also carried out both mammosphere and colony formation assays. A 2.8-fold increase in mammosphere forming ability was observed in MDA-MB-231 cells treated with BMP-4 (50 ng/ml), compared to control cells (Fig. 6e; 1.33 ± 0.17 [n = 6 wells] vs. 3.75 ± 0.36 [n = 6 wells], respectively; p < 0.0001). BMP-4 treatment also increased the cloning efficiency of MDA-MB-231 cells by approximately 2-fold, compared to control cells (Fig. 6f,g; 41% [n = 6 wells] vs. 80% [n = 6 wells], respectively; p < 0.0001). Furthermore, Noggin or GSIX pretreatment inhibited the BMP-4 mediated upregulation of the colony forming ability in these cells (Fig. 6f,g). Jagged-1 knockdown also inhibited this increase in colony formation efficiency by BMP-4-treated MDA-MB-231 cells, compared to control siRNA-transfected cells (Fig. 6h,i). Collectively, these data suggest that BMP-4 enhances the cancer stem cell traits of MDA-MB-231 cells via Notch signaling. Bone marrow-derived mesenchymal stem cells are known to be recruited into breast cancer tissue^45^ where the recruited mesenchymal stem cells increase the cancer stem cell population in breast cancer tissue and promote metastasis^45,46^. Human bone marrow-derived mesenchymal stem cells co-cultured with MDA-MB-231 cells migrated toward MDA-MB-231 cells in a manner dependent on the MDA-MB-231 cell density (Fig. 6j). BMP-4-treated MDA-MB-231 cells were better able to attract mesenchymal stem cells than control MDA-MB-231 cells (Fig. 6k). Compared with control mesenchymal stem cells, the expression of angiogenic factors such as VEGF, IL-6, or IL-8 increased in mesenchymal stem cells co-cultured with MDA-MB-231 cells, (Supplementary Fig. 11a). These angiogenic factors were also upregulated in mesenchymal stem cells incubated with media derived from non-transformed mammary epithelial cell line MCF-10A culture, compared to the cells (Supplementary Fig. 11b). However, the increase in the expression of these angiogenic factors was more remarkable in mesenchymal stem cells incubated with media derived from MDA-MB-231 cells, than with media derived from MCF-10A cells (Supplementary Fig. 11b). We also examined the effects of BMP-4 on the expression of mRNAs encoding cell cycle regulator proteins in MDA-MB-231 cells. Knockdown of Jagged-1 upregulated p21 mRNA levels in MDA-MB-231 cells in response to BMP-4 (Fig. 6l) and impaired the increase in cyclin D1 mRNA levels in BMP-4 treated MDA-MB-231 cells (Fig. 6m). We addressed the effects of BMP-4 on tumorigenic capacity *in vivo* using a xenograft model of MDA-MB-231 cells. Tumor formation was accelerated using BMP-4-treated MDA-MB-231 cells compared to control cells. More importantly, pre-treatment with γ-secretase inhibitor X (GSIX) inhibited the tumorigenesis enhanced by BMP-4 (Fig. 6n). Taken together, these results suggest that BMP-4 enhances tumorigenesis in MDA-MB-231 cells, as well as its cancer stem cell properties, via Notch activation.

### BMP-4-associated Notch signaling enhances the EMT and the cancer stem cell capacity of patients with breast carcinoma

**Figure 7:**
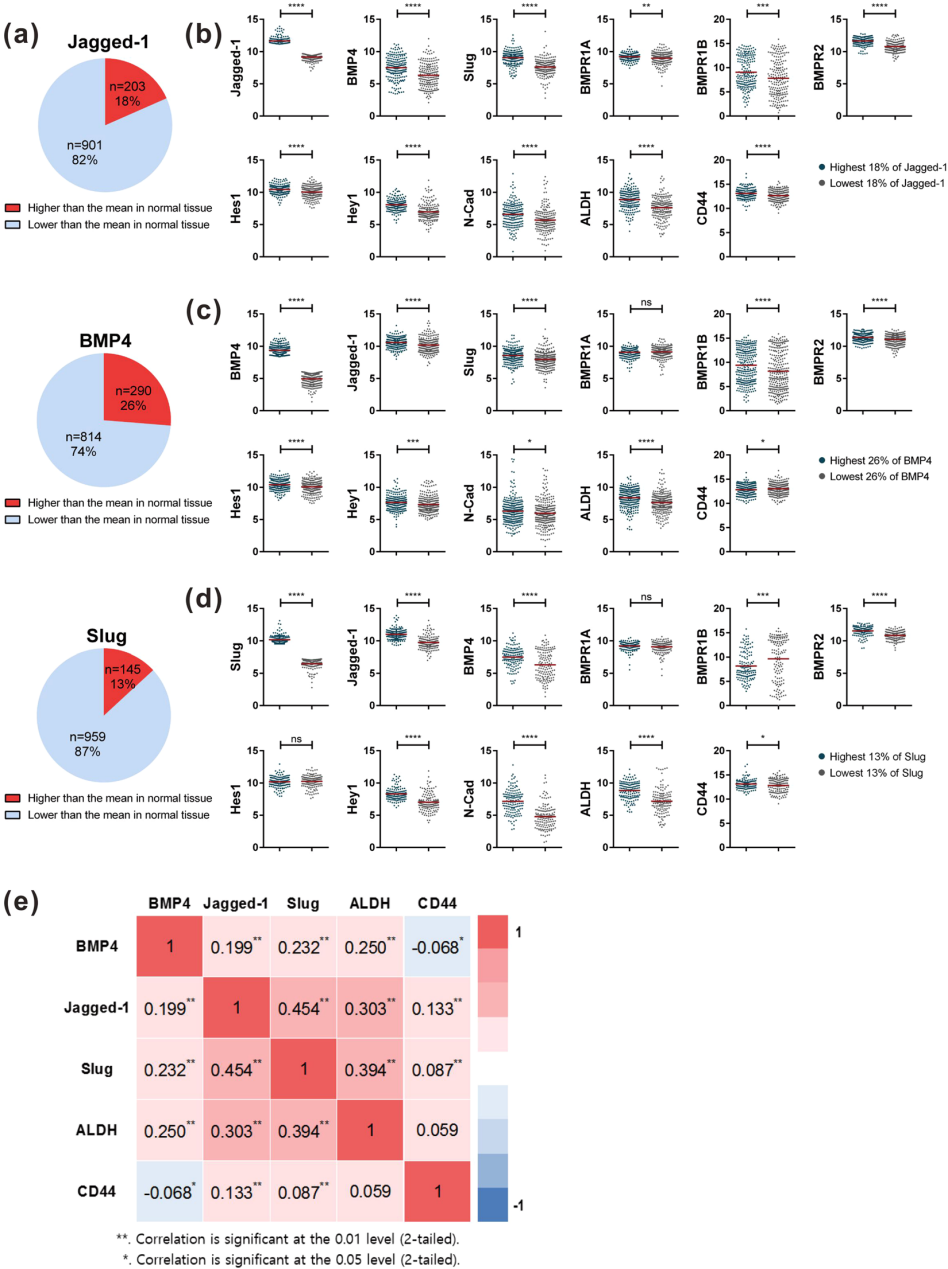
Jagged-1 expression is associated with the expression of BMP-4, EMT associated genes, and cancer stem cell marker genes in human breast cancer. (**a**) The expression of Jagged-1, BMP-4, and Slug in breast cancer compared to that in normal tissue. (**b–d**) Samples with the highest 18% (green dots) and the lowest 18% (gray dots) expression of Jagged-1 (**b**), the highest 26% (green dots) and the lowest 26% (gray dots) expression of BMP-4 (**c**), and the highest 13% (green dots) and the lowest 13% (gray dots) expression of Slug (**d**) were selected for analysis and *p*-values were calculated using Student’s *t*-test (*p < 0.05, **p < 0.01, ***p < 0.001, ****p < 0.0001, ns; not significant). Mean values are shown as red lines. (**e**) Spearman’s rank-order correlation test analysis for BMP-4, Jagged-1, Slug, ALDH, and CD44 in breast cancer. Numerical values of correlation coefficients are presented in the boxes.

Following this, we analyzed the RNA-seq database of human breast cancers from The Cancer Genome Atlas (TCGA). The expression levels of Jagged-1 and BMP-4 were not higher in breast cancer compared to those in paired normal tissue. However, an abnormal increase in Jagged-1 expression was found in more than 30% of patients with metastatic breast cancer. The expression levels of Jagged-1 and BMP-4 were higher than the baseline level (as the average level measured in paired normal tissue) in approximately 18% and 26% of breast cancer tissue samples, respectively (Fig. 7a). Genetic alterations in BMPR1A, BMPR1B, or BMPR2 were found in around 6, 7, or 5% of patients with breast cancer, respectively, with the major form of genetic alternation being an upregulation of mRNA encoding BMP receptors (data not shown). In addition, the expression of Jagged-1 significantly correlated with the expression of BMP-4, EMT markers such as Slug, and cancer stem cell makers including ALDH and CD44 (Fig. 7b–e). Moreover, the expressions of Jagged-1, BMP-4, Slug, and ALDH were reciprocally linked to each other (Fig. 7b–e). We also analyzed the microarray data of human breast cancer cell lines from the Cancer Cell Line Encyclopedia (CCLE) and found that the mesenchymal subtypes of human breast cancer cell lines^47–50^ showed higher expression levels of BMP-4, Jagged-1, Hey1, CD44, and Slug than the luminal subtypes (Supplementary Fig. 12). Collectively, these data suggest that BMP-4-mediated signaling is associated with the activation of Notch signaling in patients with breast cancer, and that Notch activation is involved in the expression of genes related to cancer stem cell properties and EMT in breast cancer cells.

## Discussion

BMP signaling has previously been demonstrated to play roles in metastasis and the maintenance of cancer stem cell properties in breast cancer^51–53^, although contradictory effects of BMP signaling have been found^1^. Notch signaling has also separately been found to be associated with bone metastasis, poor survival, tumor recurrence, and the cancer stem cell properties of breast cancer^22,23,26,27^. We have previously demonstrated that BMP-4 induced EMT in the non-transformed mammary epithelial cell line MCF-10A^8^. In our current study, we demonstrated that BMP-4 enhanced EMT and the activation of Notch signaling via a Smad4 dependent canonical mechanism in MCF-10A cells. We also showed that Notch signaling, activated by BMP-4 canonical signaling, mediated EMT and an increase in the stem cell properties of MCF-10A cells. In addition, the BMP-4-dependent activation of Notch was demonstrated to enhance cancer stem cell properties in the breast cancer cell line MDA-MB-231.

Both Notch signaling and BMP-4-mediated signaling play important roles in breast cancer. Elevated expression of Notch and Jagged-1 mRNAs is associated with poor survival and a high risk of relapse^27^. High BMP-4 expression levels are found in 25% of breast cancer patients and are associated with a high risk of tumor recurrence^6^. BMP-4 has been shown to enhance bone metastasis of breast cancer cells in a mouse xenograft model^10^ and accelerate the migration and invasion of MDA-MB-231 cells^9,11^. However, it is not clear whether BMP-4 signaling and Notch signaling cooperate with each other in order to enhance EMT, metastasis, and tumor recurrence in breast cancers. TGF-β1, another member of the TGF-β superfamily enhances EMT and bone metastasis in breast cancer cells, including MDA-MB-231 cells, via Smad4 dependent mechanisms^35^, although it plays dual roles in carcinogenesis in breast cancer, being both a tumor suppressor and a tumor promoter depending on the tumor stage^54^. Importantly, Notch signaling is required for the TGF-β1-mediated EMT of breast cancer cells^32^. In this study, our results suggest that Notch signaling is necessary for the BMP-4-mediated EMT of breast cancer cells. It also suggests that EMT induced by BMP-4 via Notch signaling is required to maintain the cancer stem cell properties in breast cancer and for the evolution of cancer stem cells. BMP-4 enhanced Notch signaling via Smad4 in MCF-10A cells, but it is still unclear how BMP-4 activates Notch signaling in breast cancer cells or how the intrinsic Notch signal-dependent EMT enhances the cancer stem cell properties of breast cancer cells (or induces the evolution/formation of cancer stem cells). Therefore, further experiments are required to identify these mechanisms.

Bone marrow-derived mesenchymal stem cells are recruited into breast cancer tissue where they are important in formation of the tumor microenvironment^45^. Mesenchymal stem cells have been shown to enhance breast cancer metastasis^45^ and increase the cancer stem cell population in breast cancer^46^. BMP-4-treated MDA-MB-231 cells had a greater ability to recruit mesenchymal stem cells *in vitro*, compared to the control MDA-MB-231 cells, although BMP-4 did not directly induce the chemotactic migration of mesenchymal stem cells. In addition, mesenchymal stem cells incubated with MDA-MB-231 culture media, or cultured indirectly with MDA-MB-231 cells using a Millicell system, expressed higher levels of pro-angiogenic factors such as VEGF, IL-6, and IL-8, compared to control mesenchymal stem cells. These results suggest that BMP-4 may enhance the tumorigenesis of MDA-MB-231 cells *in vivo* by enhancing tumor angiogenesis. Indeed, compared to control cells, tumorigenesis was upregulated in BMP-4-treated MDA-MB-231 cells. It is still uncertain what factors originate from breast cancer cells to induce the recruitment of mesenchymal stem cells from the bone marrow to breast cancer tissue. The recruited mesenchymal stem cells may induce the evolution of cancer stem cells in breast cancer or maintain cancer stem cell properties. If this is the case, functional interactions between cancer stem cells and breast cancer mesenchymal stem cells may be important in developing a means of targeting cancer stem cells in breast cancer.

## Materials and Methods

### Cell culture

MCF-10A cells were obtained from ATCC (Manassas, VA, USA) and were grown using Dulbecco’s Modified Eagle’s Medium/Nutrient Mixture F-12 (DMEM/F12, 1:1; Invitrogen, Carlsbad, CA, USA) supplemented with 5% horse serum (Invitrogen), 20 ng/ml recombinant human epidermal growth factor (EGF; R&D systems, Minneapolis, MN, USA), 0.5 μg/ml hydrocortisone (Sigma, St. Louis, MO, USA), 100 ng/ml cholera toxin (List Biological Lab, St, Campbell, CA, USA), 10 μg/ml insulin (Sigma), and 1% penicillin and streptomycin (P/S; Invitrogen). Cells were maintained at sub-confluency and passaged using 0.05% Trypsin/EDTA (Invitrogen). Only cells at passages 4–8 were used for experiments. When required, cells were serum-starved for 24 hours using DMEM/F12 supplemented with 1% P/S, prior to treatment with 50 ng/ml BMP-4 (R&D systems). MDA-MB-231 cells (Korean Cell Line Bank, Seoul, South Korea and ATCC) were cultured using DMEM/F12 (1:1) (Invitrogen) supplemented with 10% heat-inactivated fetal bovine serum (FBS; Invitrogen) and 1% P/S (Invitrogen). When required, cells were serum-starved for 24 hours using DMEM/F12 containing 1% P/S, prior to treatment with BMP-4 at 50 ng/ml for 3 days. If necessary, cells were treated with 200 ng/ml Noggin (R&D Systems) or 5 μM γ-secretase inhibitor X (GSIX; EMD Millipore, Burlington, MA, USA) for 30 minutes prior to BMP-4 treatment. Human bone marrow derived-mesenchymal stem cells (Lonza, Basel, Switzerland) were maintained in mesenchymal stem cell growth medium (MSCGM; Lonza). Human bone marrow derived-mesenchymal stem cells between passages 3 and 6 were used for all experiments. For migration assays, the mesenchymal stem cells were serum-starved using DMEM supplemented with 2% FBS, 1% L‐glutamine, and 1% P/S. All cells were maintained at 37 °C in a humidified incubator containing 5% CO_2_. Micrographic images of the cells were captured using a Nikon ECLIPSE TS 100 inverted microscope (Nikon Instruments Inc., Melville, NY, USA).

### Small interfering (siRNA) transfection

MCF-10A or MDA-MB-231 cells were seeded into 6-well plates at a density of 1.5 × 10^5^ cells or 5 × 10^4^ cells per well, respectively, and cultured for 24 hours using complete growth media without 1% P/S prior to treatment with small interfering siRNAs (siRNAs). Small interfering RNAs (10 nM) were transfected into cells using Lipofectamine RNAiMax reagent (Invitrogen) following the manufacturer’s instructions. After 12 hours of incubation, the medium was changed to serum free media and the cells cultured for a further 24 hours. Following this, the MCF-10A or MDA-MB-231 cells were treated with 50 ng/ml BMP-4 or vehicle (4 mM HCl in 0.1% BSA) for 24 hours or 72 hours, respectively. Cells were then harvested for experiments. The sequences of the Smad4 and Jagged-1 targeted siRNAs (Genolution, Seoul, South Korea) were as follows: Smad4 siRNA #1 sense, 5′-GUGUGCAGUUGGAAUGUAAUU-3′, Smad4 siRNA #1 antisense, 5′-UUACAUUCCAACUGCACACUU-3′; Smad4 siRNA #2 sense, 5′-GAGUAAUGCUCCAUCAAGUUU-3′, Smad4 siRNA #2 antisense, 5′-ACUUGAUGGAGCAUUACUCUU-3′; Jagged-1 siRNA sense, 5′-GAAUGUGAGGCCAAACCUUUU-3′, Jagged-1 siRNA antisense, 5′-AAGGUUUGGCCUCACAUUCUU-3′. The control siRNA was obtained from Santa Cruz (Dallas, TX, USA).

### Real-time PCR

Total RNA was extracted using TRIzol (Invitrogen) and cDNA was synthesized using SuperScript III Reverse transcriptase (Invitrogen) following the manufacturer’s instructions. Real-time PCR was performed using Power SYBR Green PCR Master Mix (Invitrogen). The human ribosomal protein S9 (RPS9) gene or glyceraldehyde 3-phosphate dehydrogenase (GAPDH) gene were used as an endogenous control. The primers that were used are listed in Supplementary Table 1.

### Western blot analysis

Cells were seeded into 6-well plates using complete growth media. Cells were then serum-starved and treated with BMP-4 (50 ng/ml) for the indicated times. When required, cells were pretreated with Noggin (200 ng/ml) for 30 minutes. In order to obtain total cell lysates, the cells were washed twice with ice-cold phosphate buffered saline (PBS). Following this, 400 μl of 2 × SDS buffer (120 mM Tris-Cl [pH 6.8], 4% [w/v] SDS, 0.02% [w/v] bromophenol blue, 20% glycerol, 10% β-mercaptoethanol) was added to the cells for 5 min at room temperature. The cell lysate was collected and proteins were denatured by incubating at 95 °C for 10–15 minutes. Western blotting of proteins in the cell lysate was performed using standard procedures^55^. Primary antibodies against phosphorylated Smad1/5/9 (1:1,000, Cell Signaling Technology, Beverly, MA, USA), fibronectin (1:10, culture supernatant of clone HFN7.1 hybridoma, ATCC)^56^, laminin (1:1,000, Novus Biologicals, Centennial, CO, USA), Slug (1:1,000, Cell Signaling Technology), Jagged-1 (1:1,000, Cell Signaling Technology), Hes1 (1:1,000, Cell Signaling Technology), CD44 (1:1,000, Cell Signaling Technology), Smad4 (1:1,000, Cell Signaling Technology), Smad6 (1:1,000, Thermo Fisher Scientific, Waltham, MA, USA), and α-tubulin (1:20,000, Sigma) were used for immunoblotting. If necessary, band densities were measured using ImageJ software (NIH, Bethesda, MD, USA).

### Immunocytochemistry

MCF-10A cells were seeded on fibronectin (1 μg/ml, Sigma Aldrich) -coated covers at a density of 3 × 10^4^ per well in a 24 well plate using complete growth media. The media were changed to serum free media after 24 hours, following which the cells were incubated for another 24 hours. The cells were then treated with BMP-4 or vehicle for 24 hours. After fixing the cells with 4% formaldehyde (Sigma) for 10 minutes on ice, standard procedures^55^ were followed for immunostaining using the following primary antibodies: anti-N-cadherin (1:100; Invitrogen), anti-E-cadherin (1:100; BD Biosciences, Franklin Lakes, NJ, USA), anti-β-catenin (1:100; Bethyl Laboratories, Montgomery, TX, USA), or anti-ZO-1 (1:100; Invitrogen). The following secondary antibodies (Invitrogen) were used at a 1:500 dilution: anti-mouse IgG-Alexa 488, anti-mouse IgG-Alexa 546, anti-rabbit IgG-Alexa 488, anti-rabbit IgG-Alexa 546. When necessary, actin was stained with Alexa Fluor 546-tagged phalloidin (1:1,000; Invitrogen). The samples were mounted using ProLong Gold antifade mounting solution containing 4′,6-diamidino-2-phenylindole (DAPI; Invitrogen). Images were then captured using a Zeiss LSM 700 confocal microscope (Zeiss, Oberkochen, Germany).

### Mammosphere assay

Sub-confluent MCF-10A cells were detached and washed twice with serum free media. After the final wash, the cells were suspended in assay media consisting of DMEM/F12 supplemented with B27 (1:50, Invitrogen), 20 ng/ml EGF (R&D systems), 10 µg/ml insulin (Sigma), 20 ng/ml mouse recombinant FGF basic (bFGF, R&D Systems), and 1% P/S. Cells (8,000) were seeded in each well of a 24-well low-attachment plate using 0.5 ml of assay media (Corning Inc., NY, USA) and incubated for 9 days. Fresh medium (0.1 ml) was added to each well every 3 days. When required, BMP-4 (50 ng/ml) or vehicle was added to the cell suspension before seeding. In some experiments, Noggin (200 ng/ml) or GSIX (5 μM) was added prior to BMP-4 treatment. After the 9 day incubation period, spheres (n = 30~56 spheres/group) were visualized and analyzed. The number of spheres with a diameter ≥100 μm was determined. For MDA-MB-231 cells, 2,000 cells were seeded in 0.8 ml of mammosphere assay media. After the 10 day incubation period, spheres (n = 71~78 spheres/group) were visualized and analyzed. The number of MDA-MB-231 spheres with a diameter ≥200 μm was then determined.

### Colony formation assay

MDA-MB-231 cells (50 cells) were plated in a well of a 6-well plate using complete culture media. After incubation for 10 days, the plates were washed with PBS and fixed with methanol for 10 minutes, Following this, the plates were stained using 0.5% methyl violet (Sigma) for 1 hour. Pictures of colonies were obtained and the numbers of colonies containing more than 50 cells were counted.

### Cell-conditioned media

MCF-10A or MDA-MB-231 cells were cultured until confluent using complete culture media. Following this, the cells were incubated using serum free culture media for 3 days. At the time of collection, cellular debris were removed using a filter (0.22 μm pore size) and aliquots were frozen at −70 °C until utilization.

### Migration assay

Millicell culture plate inserts (8 μm pore size; EMD Millipore) were coated with type I collagen (5 μg/ml; Nitta Gelatin Inc.). Serum starved human bone marrow derived-mesenchymal stem cells were seeded at a density of 3 × 10^4^ cells/cm^2^ into the upper chamber of the inserts and incubated for 6 hours. Following this, cell-conditioned media or control media were added to the lower chamber of each insert. For the migration assay using an indirect co-culture system with MDA-MB-231 cells, the inserts were incubated in 6-well plates seeded with MDA-MB-231 cells. After 12 hours of incubation, the inserts were fixed using 4% paraformaldehyde in phosphate-buffered saline (PBS) for 10 minutes. Following this, mesenchymal stem cell nuclei were stained using DAPI (Invitrogen) for 7 mins and the membrane of the inserts was mounted on slides using ProLong Gold antifade mounting solution (Invitrogen). Cells on either the lower or upper sides of the membrane were imaged by confocal microscopy using five randomly selected areas in each insert (Zeiss LSM700). The cell numbers in each image were counted using Adobe Photoshop CS6 and the number of migrated cells as a percentage of the total cells on both sides of the insert was calculated.

### Subcutaneous xenograft experiments

All procedures were approved by the animal experiment ethics committee of Kyung Hee Hospital Medical Center (KHMC-IACUC-15-037) and performed under the Institutional Animal Care and Use Committee (IACUC) guidelines. Female 7-week-old-BALB/c nude mice (DBL, Seoul, South Korea) were subcutaneously transplanted with MDA-MB-231 cells treated or not with BMP-4 for 72 hours. If necessary, cells were treated with 5 μM γ-secretase inhibitor X (GSIX) for 30 minutes prior to BMP-4 treatment. MDA-MB-231 cells were suspended in PBS and mixed in a 1:1 ratio with Matrigel (BD Biosciences). The mixture (2 × 10^5^ cells in 0.1 ml) was injected subcutaneously on the right flank of each mouse. After 9 weeks of transplantation, the mice were sacrificed and tumors were harvested for weighing.

### Statistical analysis

Quantitative data are presented as the mean ± standard deviation (SD). The significance of differences between two groups, or between multiple groups, was analyzed using an unpaired Student’s *t*-tests or a one-way analysis of variance (ANOVA), respectively. Differences with *p* values of less than 0.05 were considered significant. Statistical analyses were performed using GraphPad version 5.01 (GraphPad Software, Inc. CA, USA; http://www.graphpad.com).

## Supplementary information

Supplementary Information

## Data Availability

Data analyzed during this study are included in this published article. Any additional information are available from the corresponding author on reasonable request.
