# Supplementary material for: BMP-4 enhances epithelial mesenchymal transition and cancer stem cell properties of breast cancer cells via Notch signaling
Source: Sci Rep. 2019 Aug 13;9:11724. doi: 10.1038/s41598-019-48190-5 (PMC6692307; doi:10.1038/s41598-019-48190-5)
Supplement: Supplementary file 1 — Supplementary Information [file 41598_2019_48190_MOESM1_ESM.pdf]

Supplementary Information

BMP-4 enhances epithelial mesenchymal transition and cancer stem cell properties of breast cancer cells via Notch signaling

Sanghyuk Choi[1,†], Jinyeong Yu[1,†], Aran Park[1], Maria Jose Dubon[1], Jungbeom Do[2], Youngjae Kim[2], Donghyun Nam[2], Jinok Noh[2], Ki-Sook Park[2,3,4,*]

[1]Graduate School of Biotechnology, Kyung Hee University, Yongin 17104, Korea

[2]Department of Biomedical Science and Technology, Graduate School, Kyung Hee University, Seoul 02447, Korea

[3]East-West Medical Research Institute, Kyung Hee University, Seoul 02447, Korea

[4]College of Medicine, Kyung Hee University, Seoul 02447, Korea

*kisookpark@khu.ac.kr

[†]these authors contributed equally to this work

Gene	Primer sequence (5'- 3')<br>Forward-Reverse	Length	T_m (℃)
BMP4	5'-GATCCACAGCACTGGTCTTG-3'	20	57.4
	5'-GGGATGCTGCTGAGGTTAAA-3'	20	55.4
Cyclin D1	5'-GCCACAGATGTGAAGTTCATT-3'	21	54.1
	5'-GGGTCACACTTGATCACTCT-3'	20	55.4
CD44	5'-TTTGAATATAACCTGCCGCTTTG-3'	23	54.9
	5'-GGTGTTGGATGTGAGGATGT-3'	20	55.4
Fibronectin	5'-AAACTTGCATCTGGAGGCAAACCC-3'	24	60.4
	5'-AGCTCTGATCAGCATGGACCACTT-3'	24	60.4
Hey1	5'-CGGCTCTAGGTTCCATGTCC-3'	20	59.5
	5'-GCTTAGCAGATCCTTGCTCCA-3'	21	58.2
Hes1	5'-AGGCGGACATTCTGGAAATG-3'	20	55.4
	5'-CGGTACTTCCCCAGCACACTT-3'	21	59.8
IL-6	5'-TTCAATGAGGAGACTTGCCTGG-3'	22	58.4
	5'-CTGGCTTGTTCCTCACTACTCT-3'	22	58.4
IL-8	5'-ATAAAGACATACTCCAAACCTTTCCAC-3'	27	58.1
	5'-AAGCTTTACAATAATTTCTGTGTTGGC-3'	27	56.4
Jagged-1	5'-ATGGGCCCCGAATGTAACAG-3'	20	57.4
	5'-TCCCACAGTAATTGAGATCTTTGT-3'	24	55.1
Laminin-5	5'-AGGCTGTCCAACGAAATGGG-3'	20	57.4
	5'-GGAGCTGTGATCCGTAGACCA-3'	21	60.3
Nanog	5'-AGTCCCAAAGGCAAACAACCCACTTC-3'	26	62.4
	5'-TGCTGGAGGCTGAGGTATTTCTGTCTC-3'	27	63.8
N-cadherin	5'-CATCCCTCCAATCAACTTGC-3'	20	55.4
	5'-ATGTGCCCTCAAATGAAACC-3'	20	53.4
p21	5'-GCAGACCAGCATGACAGATTT-3'	21	56.2
	5'-GGATTAGGGCTTCCTCTTGGA-3'	21	58.2
Slug	5'-AGATGCATATTCGGACCCAC-3'	20	55.4
	5'-CCTCATGTTTGTGCAGGAGA-3'	20	55.4
Smad4	5'-ATCTATGCCCGTCTCTGGAGGT-3'	22	60
	5'-CAGGAATGTTGGGAAAGTTGGC-3'	22	58.4
Smad6	5'-CCTACCGTGTGCTGCAACC-3'	19	59.7
	5'-GTGGAATCGGACAGATCCAGTG-3'	22	60
VEGF	5'-CTGCTCTACCTCCACCATGC-3'	20	59.5
	5'-AGCTGCGCTGATAGACATCC-3'	20	57.4
GAPDH	5'-CAGCCTCAAGATCATCAGCA-3'	20	55.4
	5'-TGTGGTCATGAGTCCTTCCA-3'	20	55.4
RPS9	5'-CTGACGCTTGATGAGAAGGAC-3'	21	58.2
	5'-CAGCTTCATCTTGCCCTCAT-3'	20	55.4

Supplementary Table 1. Primers used for target genes

Choi et al., Supplementary Figure 1

a

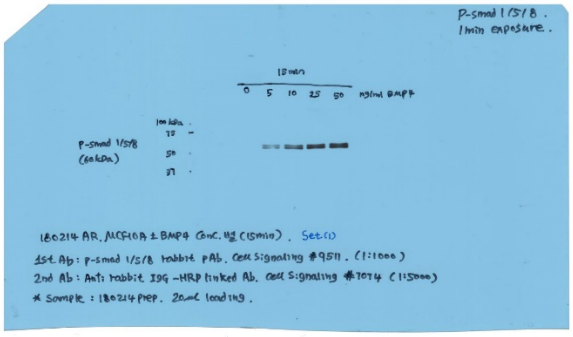

b

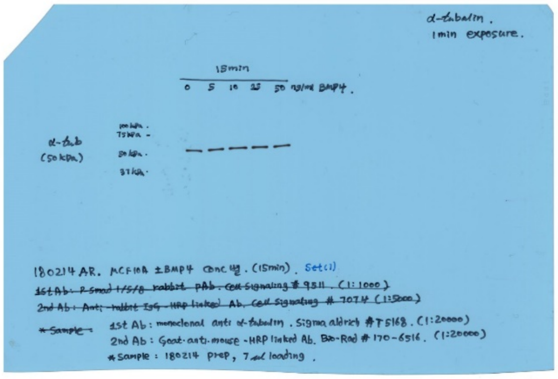

C

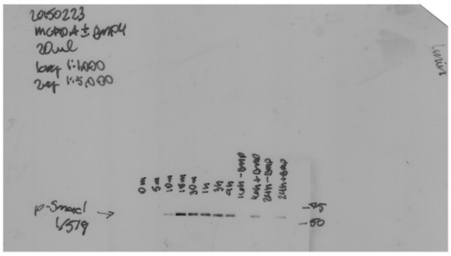

d

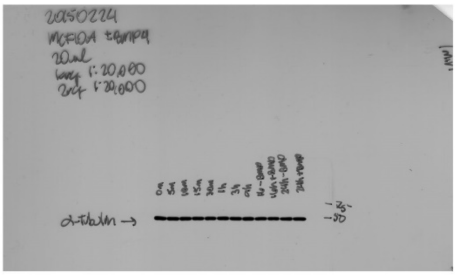

Supplementary Figure 1. Full-length unedited blots corresponding to Figure 1a and c showing expression of pSmad1/5/9 and α-tubulin (α-Tub) in the panels (**a,b**) and (**c,d**), respectively.

a

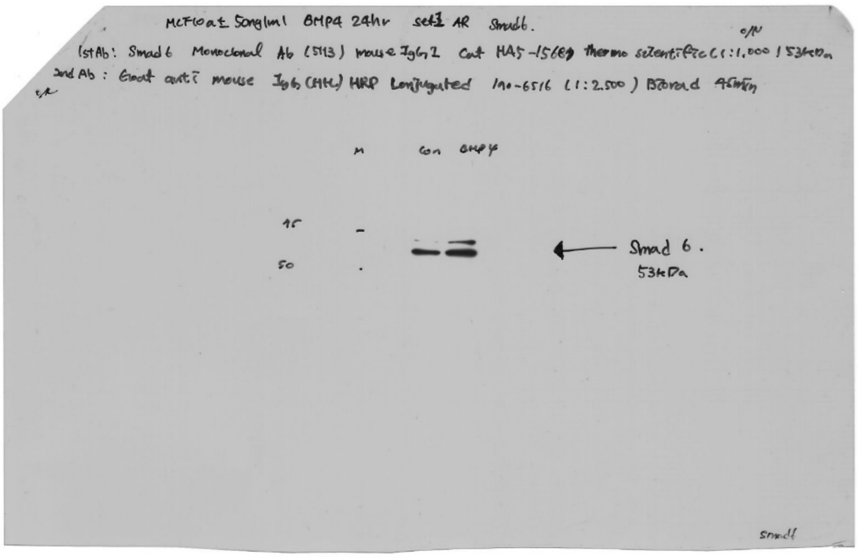

b

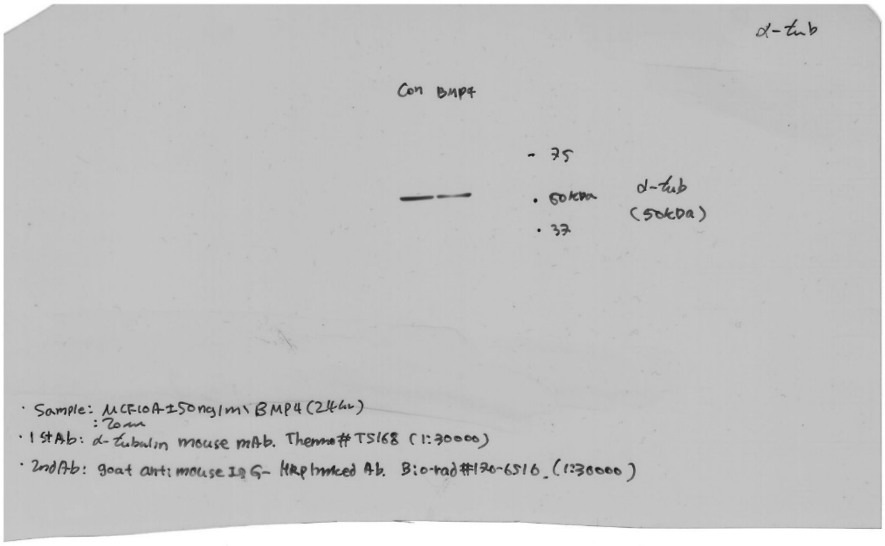

Supplementary Figure 2. Full-length unedited blots of Smad6 and α-tubulin. (**a**) Western blot analysis showing Smad6 expression in the MCF-10A cell extract treated with BMP-4 (50 ng/ml) or vehicle (CON) for 24 hours. (**b**) α-Tubulin was used as an internal control.

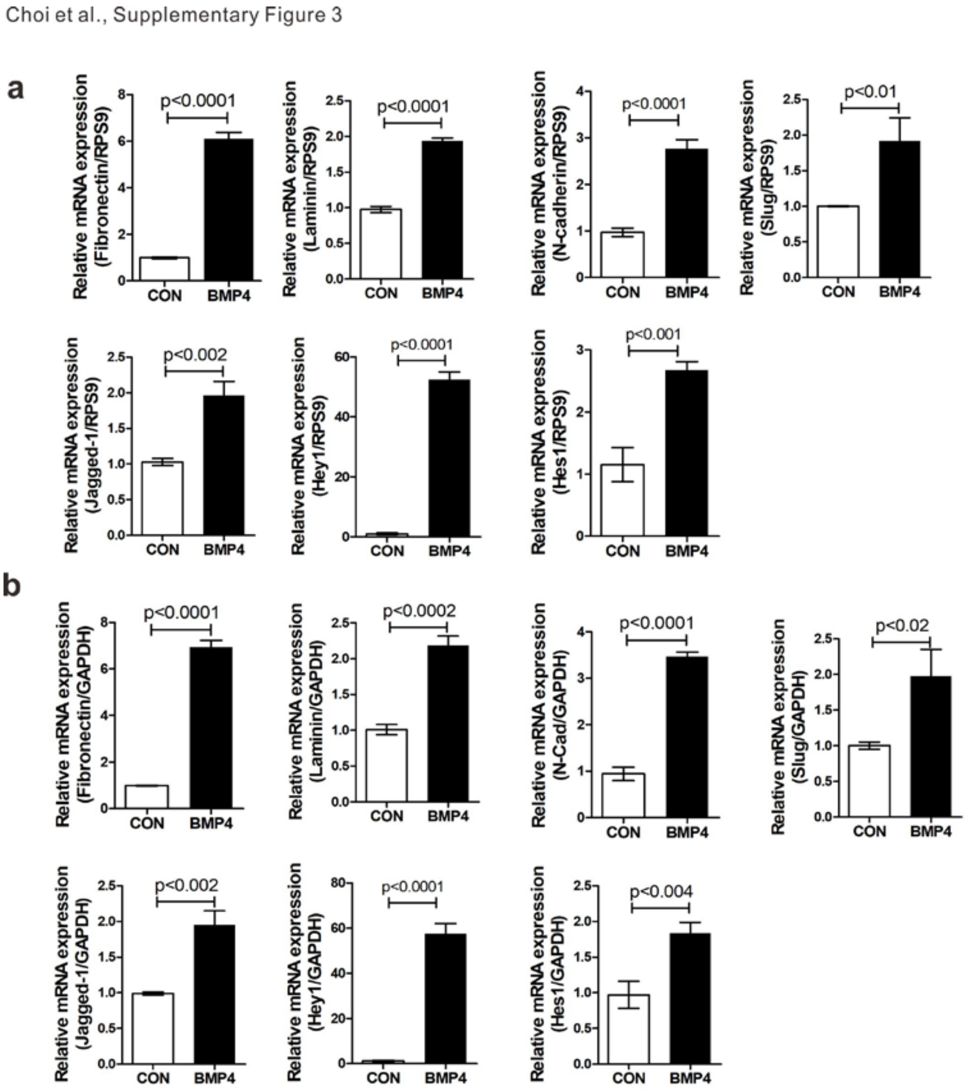

Supplementary Figure 3. Real-time PCR analysis to check mRNA expression of EMT-associated genes and Notch target genes in MCF-10A cells treated with BMP-4 (50 ng/ml) or vehicle (CON) for 24 hours. The human ribosomal protein S9 gene (**a**; RPS9) and glyceraldehyde 3-phosphate dehydrogenase gene (**b**; GAPDH) were used as endogenous controls, respectively. Data are presented as mean ± SD and *p*-values were calculated using a Student's *t*-test.

Choi et al., Supplementary Figure 4

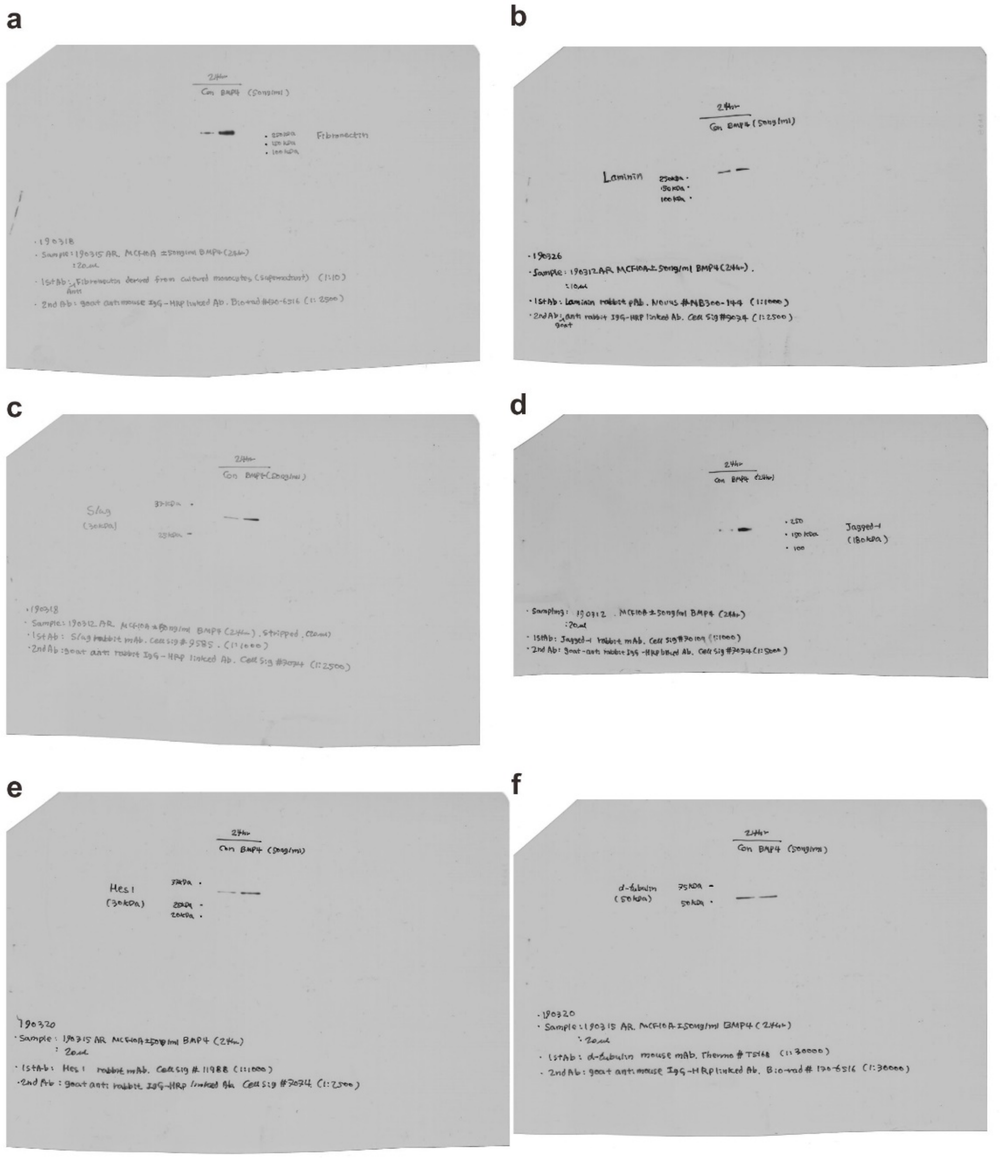

Supplementary Figure 4. Full-length unedited blots corresponding to Figure 1h. Full-length unedited blots for fibronectin (**a**), laminin (**b**), Slug (**c**), Jagged-1 (**d**), Hes1 (**e**) and α-tubulin (**f**) in MCF-10A cells treated with BMP-4 (50 ng/ml) or vehicle (CON) for 24 hours.

a

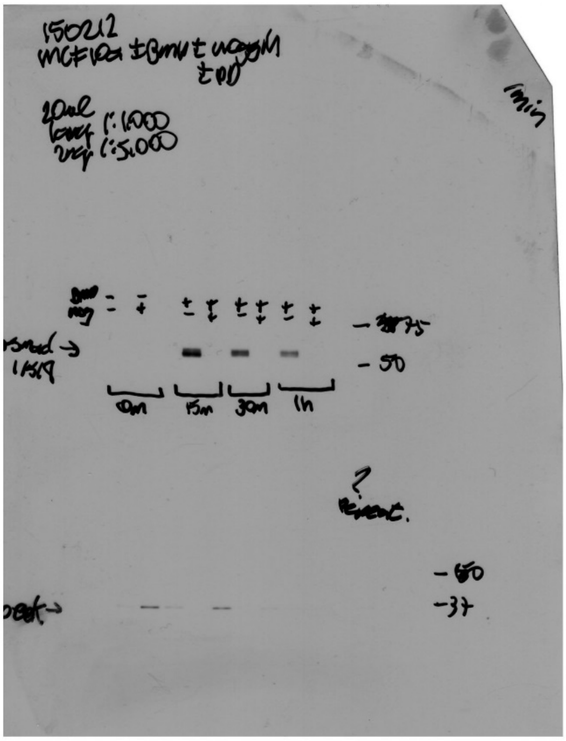

b

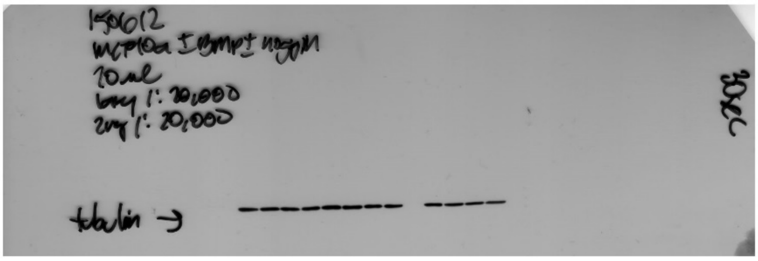

Supplementary Figure 5. Full-length unedited blots corresponding to Figure 2a. Full unedited blots for pSmad1/5/9 (**a**) and α-tubulin (**b**) of Figure 2a.

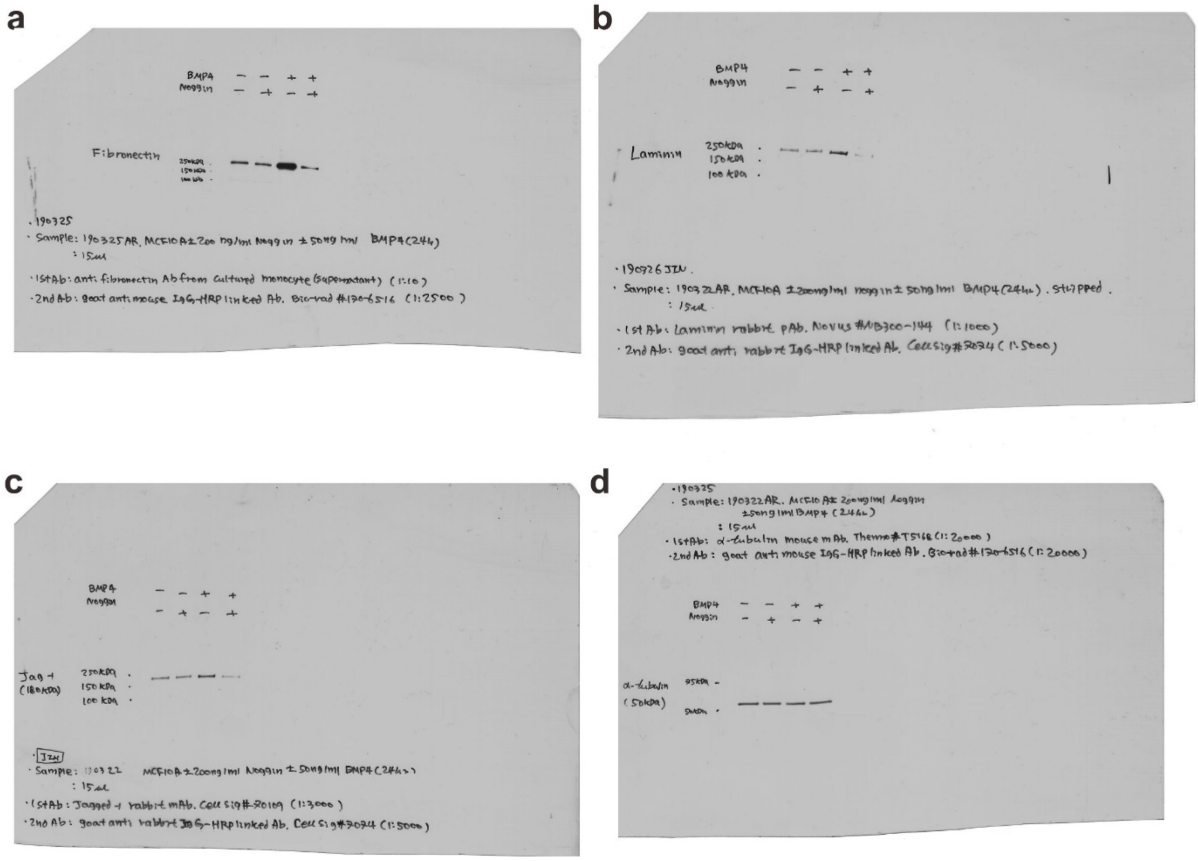

Supplementary Figure 6. Full-length unedited blots corresponding to Figure 2e. Full-length unedited blots for fibronectin (**a**), laminin (**b**), Jagged-1 (**c**), and α-tubulin (**d**) in MCF-10A cells treated with BMP-4 (50 ng/ml) or vehicle (CON) for 24 hours.

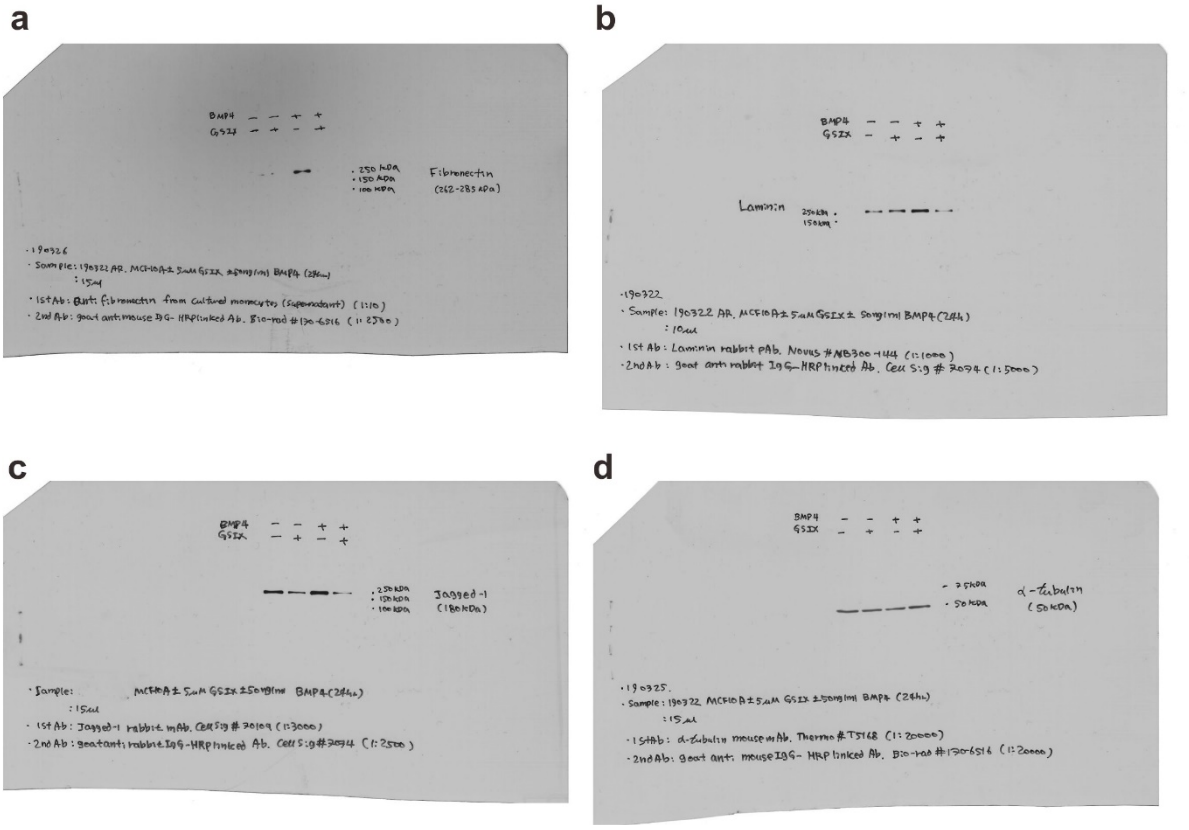

Supplementary Figure 7. Full-length unedited blots corresponding to Figure 3e. Full-length unedited blots for fibronectin (**a**), laminin (**b**), Jagged-1 (**c**), and α-tubulin (**d**).

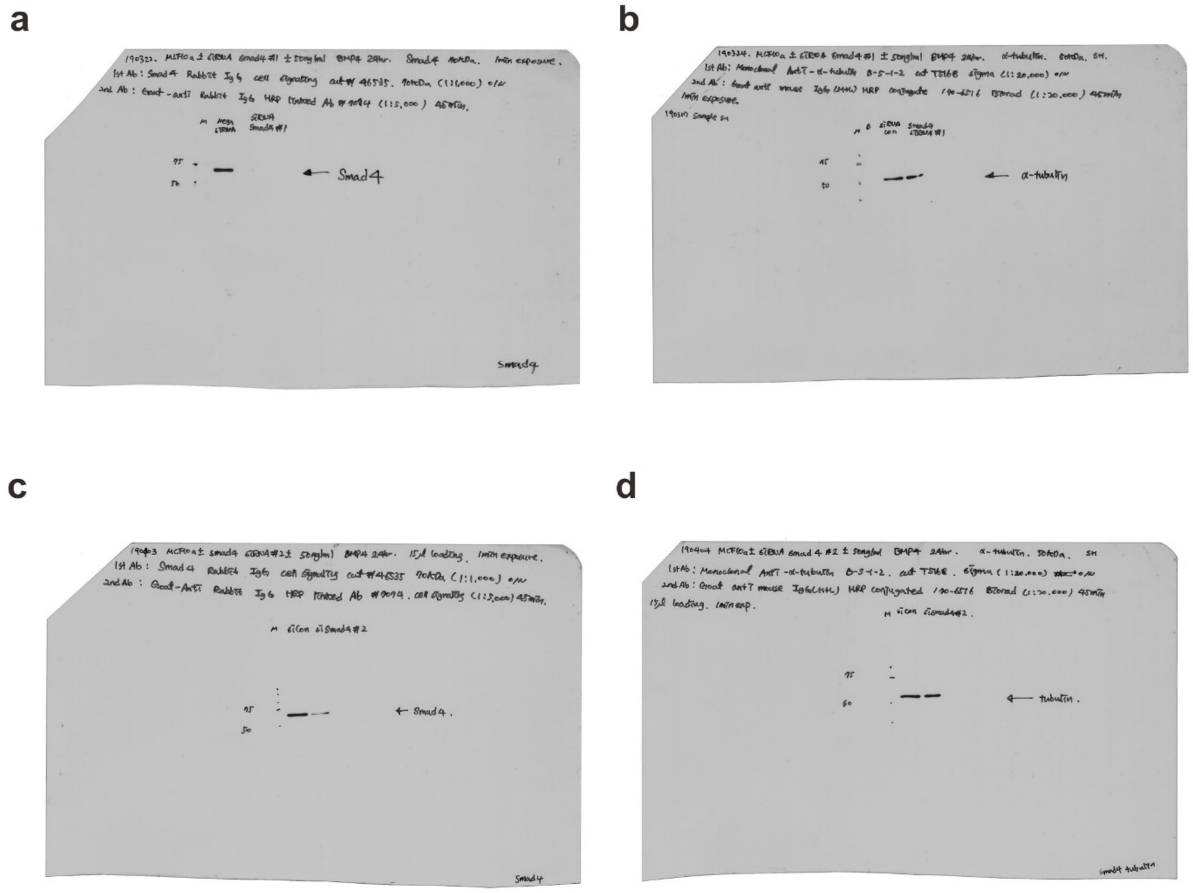

Supplementary Figure 8. Full-length unedited blots corresponding to Figure 4b.

Full-length unedited blots for Smad4 (**a,c**) and α-tubulin (**b,d**).

a

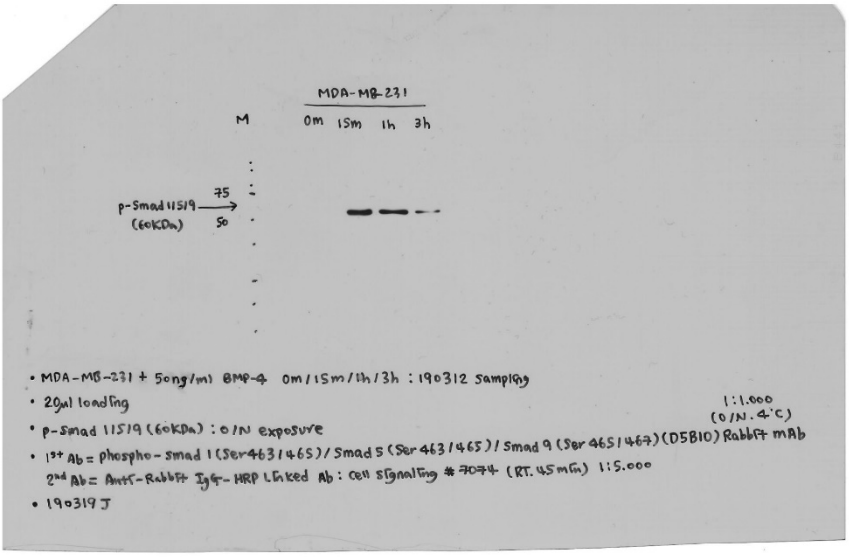

b

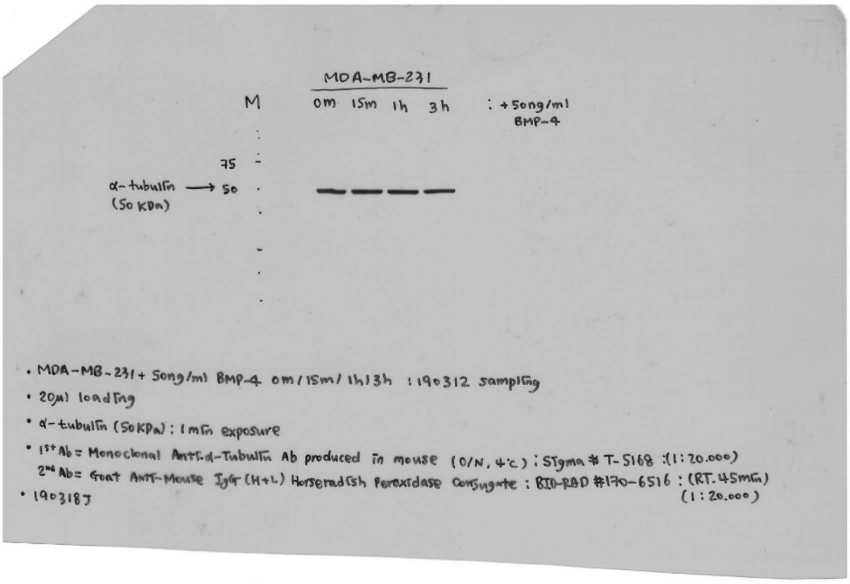

Supplementary Figure 9. Full-length unedited blots corresponding to Figure 6a. Full-length unedited blots for phosphorylated Smad1/5/9 proteins (pSmad1/5/9) (**a**) and α-tubulin (**b**) in MDA-MB-231 cells treated with BMP-4 (50 ng/ml) for the indicated time durations.

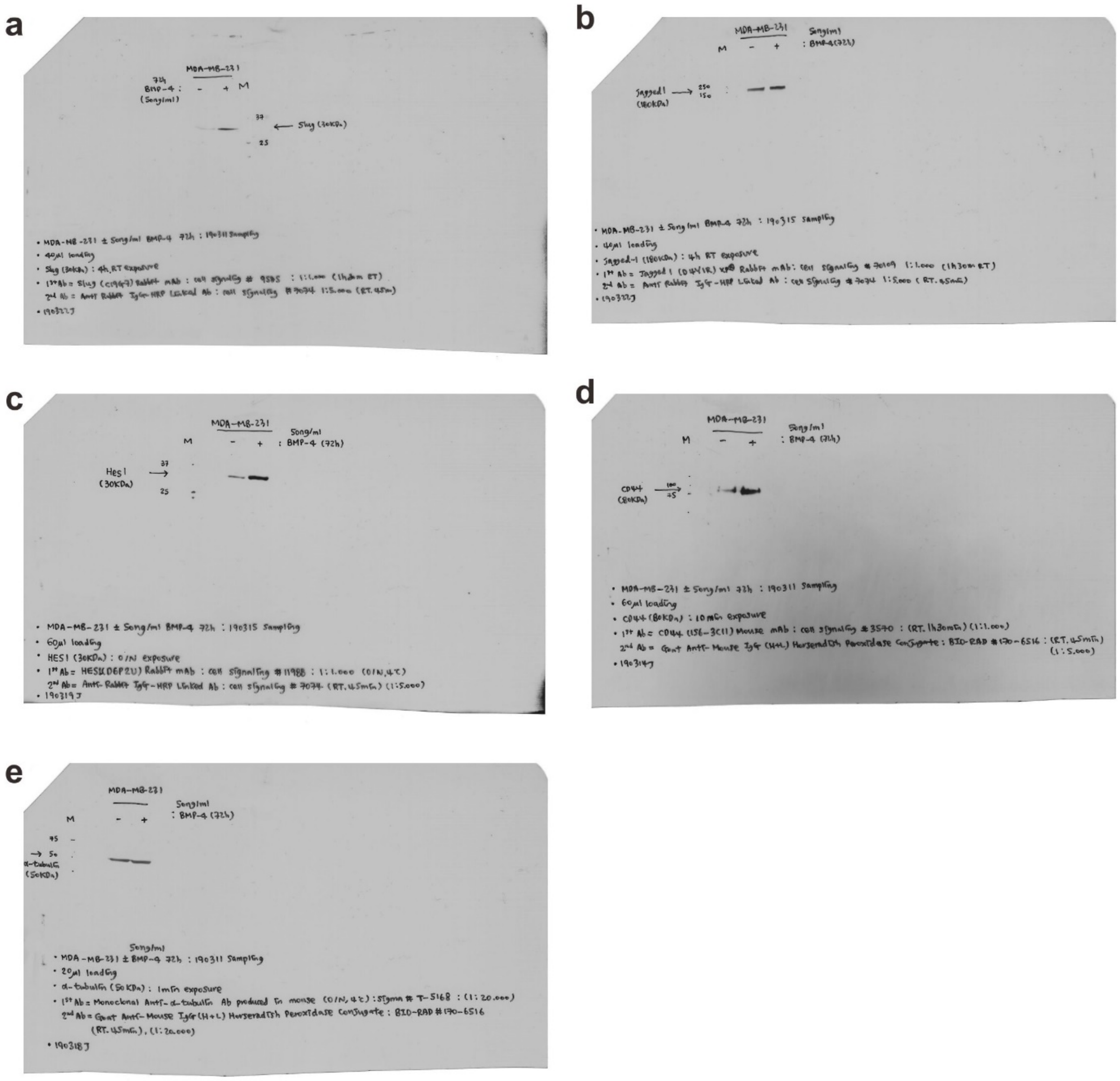

Supplementary Figure 10. Full-length unedited blots corresponding to Figure 6c. Full-length unedited blots for Slug (**a**), Jagged-1 (**b**), Hes1 (**c**), CD44 (**d**), and α-tubulin (**e**) in MDA-MB-231 cells treated with BMP-4 (50 ng/ml) or vehicle (CON) for 72 hours.

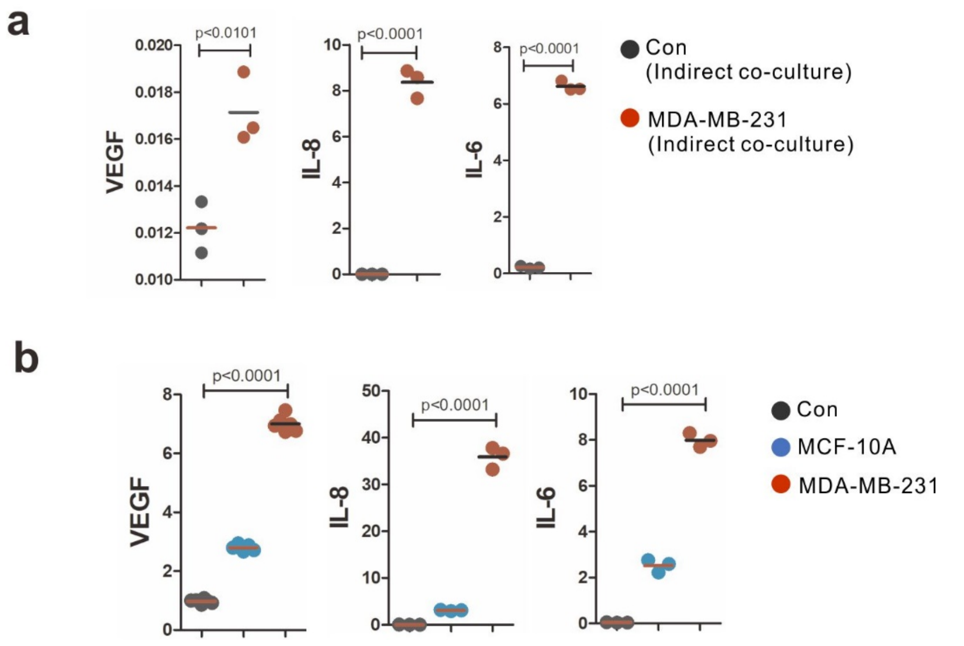

Supplementary Figure 11. Mesenchymal stem cells increase the expression of pro-angiogenic factors in response to MDA-MB-231 cells. (**a**) Real-time PCR analysis of the expression of VEGF, IL-8, and IL-6 in human bone marrow derived-mesenchymal stem cells indirectly co-cultured with MDA-MB-231 cells (red circles) or with MDA-MB-231 complete culture media (gray circles). (**b**) Real-time PCR analysis of the expression of VEGF, IL-8, and IL-6 in human bone marrow derived-mesenchymal stem cells cultured with MCF-10A-conditioned media (blue circles), MDA-MB-231-conditioned media (red circles) or control media (gray circles). Human ribosomal protein S9 (RPS9) was used as an internal control. The red lines indicate mean values and *p*-values were calculated using a Student's *t*-test.

Choi et al., Supplementary Figure 12

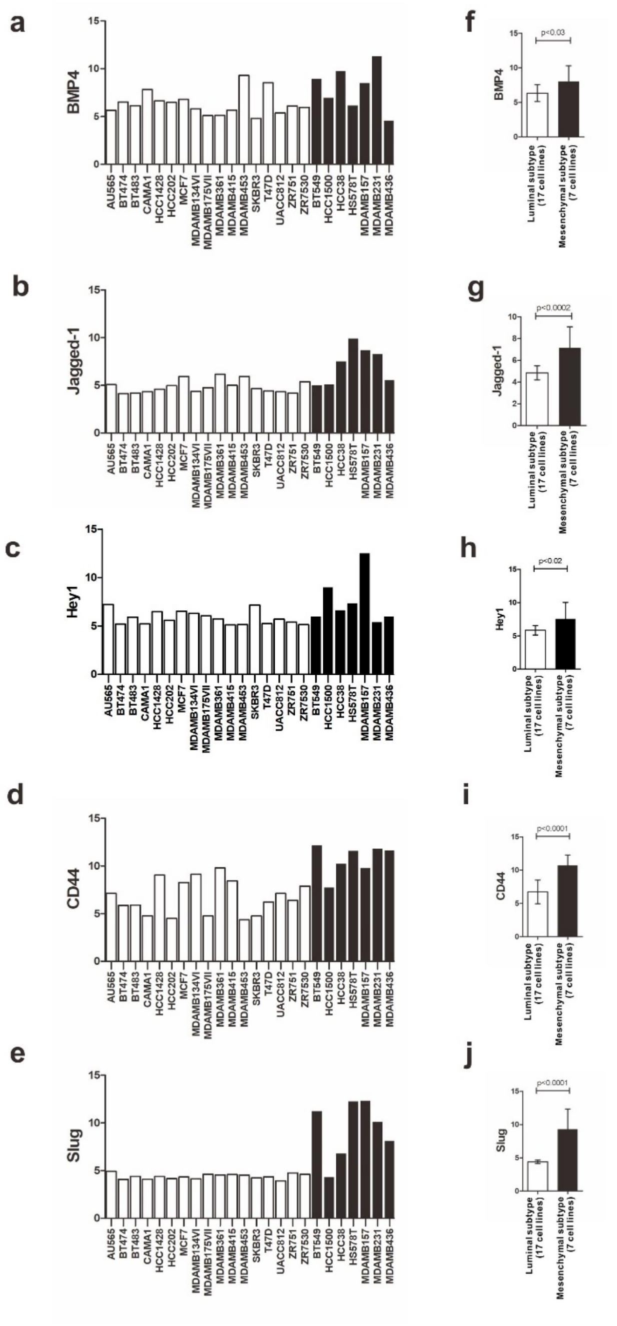

Supplementary Figure 12. The expression levels of various genes in human breast cancer cell lines. The expression of BMP-4 (**a**), Jagged-1 (**b**), Hey1 (**c**), CD44 (**d**), and Slug (**e**) in 17 luminal subtype cell lines (white bars) and 7 mesenchymal subtype cell lines (black bars). (**f-j**) The statistical analysis of the expression levels of

the genes in each subtype of human breast cancer cell lines were performed. Data are presented as the mean ± SD and *p*-values were calculated using a Student's *t*-test.
